# Characterization of Crack Evolution and Subsurface Damage in AA7075-T6, Anodized AA6061-T6, and Al-15 vol.% SiC T4 Composite Under Wear and Indentation Loading

**DOI:** 10.3390/ma19183957

**Published:** 2026-09-17

**Authors:** Emmanuel Sey, Syed Ali Husnain, George Jarjoura, Zoheir N. Farhat

**Affiliations:** Department of Mechanical Engineering, Dalhousie University, Halifax, NS B3H 4R2, Canada; em445321@dal.ca (E.S.); hu408941@dal.ca (S.A.H.); george.jarjoura@dal.ca (G.J.)

**Keywords:** AA7075-T6, anodized AA6061-T6, Al-15 vol.% SiC T4 composite, multipass scratch, hertzian contact, nanoindentation

## Abstract

This study investigated the deformation behavior and damage evolution of AA7075-T6, Anodized AA6061-T6-T6 (oxide layer with an average thickness of 8.71 µm), and Al-15 vol.% SiC-T4 (particle size range of 3.1–8.3 µm) under scratch, Hertzian-type indentation, and nanoindentation loading conditions. Through controlled mechanical testing, the materials’ responses to varying test conditions were characterized in terms of penetration depth and width, resistance to deformation, and cracking mechanisms on surface and sub-surface. Results revealed that Al-15 vol.% SiC T4 exhibited the highest stability and scratch resistance due to the reinforcing effect of SiC particles, while AA7075-T6 demonstrated moderate strength with noticeable plastic deformation. Anodized AA6061-T6 showed enhanced surface hardness post processing but increased susceptibility to brittle cracking and coating delamination under higher loads and reciprocating passes. Findings indicated a transition from load-controlled to structure-controlled behavior at elevated forces, influenced by strain hardening and microstructural constraints. Hertzian-type indentation further highlighted differences in subsurface damage and crack propagation patterns among the materials. Holistically, the study established a clear correlation between microstructural features and mechanical performance, guiding material selection and surface engineering for improved wear and contact damage resistance.

## 1. Introduction

Aluminum (Al) and its alloys are extensively used in aerospace, automotive, and structural systems owing to their exceptional strength-to-weight ratio, corrosion resistance, and manufacturability [1]. As their use in tribological and contact-intensive components continues to expand, their long-term performance is increasingly governed by their susceptibility to localized damage processes that arise under service conditions involving contact loading, frictional wear, or repeated mechanical interaction. These conditions can trigger wear-induced abrasion, indentation-driven plastic deformation, and the initiation and growth of cracks beneath contact interfaces, ultimately leading to subsurface damage accumulation. Such degradation mechanisms are especially critical in components exposed to cyclic contact, fretting, impact loading, or severe environments, where crack evolution and internal damage often progress silently until they manifest as significant loss of function or catastrophic failure.

Previous research on subsurface deformation in Al alloys under sliding wear and indentation has shown that crack formation often initiates at depths significantly below the indented or worn surface. Early studies by Bhattacharya et al. and Schwarzenböck et al. [2,3] demonstrated that particle-matrix decohesion, localized shear banding, and microvoid formation are dominant mechanisms under high contact stresses. More recent investigations using in situ and ex situ X-ray imaging techniques [4] have confirmed that repeated contact accelerates the growth and interconnection of these internal defects, eventually causing crack propagation toward the surface and contributing to delamination wear.

Furthermore, an investigation by M. Ruiz-Andrés et al. [5] on the wear behavior of Al alloys at extremely slow sliding speeds aimed at determining whether conventional wear processes remain valid when sliding speeds fall below 0.01 m/s, using dry block-on-ring tests on Al-Mg, Al-Mg-Si, and Al-Zn-Cu alloys against an AISI 52100 steel ring further describes the evolution of wear mechanisms under low-velocity conditions. It was found that at speeds above 0.01 m/s, all alloys displayed mild wear governed by oxidation and delamination, consistent with established wear regimes for Al. However, when the sliding speed dropped below 0.01 m/s, a marked increase in both wear rate and friction coefficient was observed across all alloys, signaling the onset of a different wear mechanism likely linked to reduced oxide-film stability and changes in subsurface stress distribution. The study demonstrates that sliding velocity exerts a stronger influence on wear behavior than alloy composition and highlights the need for deeper mechanistic understanding and investigation into speed-related applications, offering valuable theoretical context for subsurface damage and crack evolution studies.

Additional context is provided by the work of Torabian et al. [6], who investigated the wear characteristics of Al-Si alloys containing 2–20 wt.% Si using a pin-on-disc test configuration at room temperature. Their study examined how alloy composition, sliding distance, sliding speed, and applied load influence wear behavior across a broad operational range. The results showed that wear rate is highly sensitive to both alloy chemistry and testing conditions, with Si content playing a central role in determining performance. As Si content increased, the wear rate decreased significantly and the load-bearing capacity of the alloys improved, reflecting the strengthening and hardening effects of the eutectic Si phase. The researchers also observed that the dominant wear mechanism shifted depending on Si level and testing parameters, indicating that microstructural composition directly governs the transition between mild and more severe wear regimes. Other studies, including foundational work by David Tabor [7] established that indentation hardness in metals reflects resistance to plastic deformation and the associated energy absorbed during loading. This was complemented by classical contact mechanics developed by K.L. Johnson [8], which describes stress distribution beneath indenters and the role of subsurface shear stresses in crack initiation and propagation.

Recent nanoindentation studies have shown that inverse interpretation from load–displacement data may not be unique [9]. Orientation-dependent responses have also been reported for single-crystal materials and for Berkovich indentation [10,11]. These studies support cautious interpretation of shallow-depth hardness and modulus values.

Despite extensive investigations into the wear and contact damage behavior of Al-based materials, the existing literature predominantly reports surface-level wear metrics such as mass loss, friction coefficient, and groove morphology under fixed, single-pass loading conditions. Systematic studies that track the progressive evolution of subsurface crack initiation, propagation, and damage accumulation across increasing load magnitudes and reciprocating cycles remain scarce. Moreover, direct comparative characterization of mechanistically distinct Al-based systems, monolithic high-strength alloys, ceramic-coated variants, and particle-reinforced composites, under identical, controlled contact conditions has not been comprehensively reported, limiting the ability to draw microstructure-informed conclusions about damage tolerance and material selection for tribological applications. To address this gap, three Al-based material systems were strategically selected to represent fundamentally different damage accommodation mechanisms: AA7075-T6, a precipitation-hardened alloy that deformed primarily through ductile plastic flow governed by dislocation-precipitate interactions; anodized AA6061-T6, a coated system in which a hard, brittle Al_2_O_3_ surface layer introduced a mechanical mismatch with the ductile substrate, thereby altering crack initiation and coating-substrate interfacial behavior; and an Al-15 vol.% SiC T4 metal matrix composite, in which hard ceramic reinforcement particles imposed heterogeneous stress fields and promoted particle-matrix debonding and third-body abrasion mechanisms that were absent in the monolithic alloys. Together, these three systems spanned the principal surface engineering and strengthening strategies employed in structural and tribological Al components, enabling a mechanistically meaningful cross-comparison under a unified experimental framework.

Specifically, this study addresses the following questions that prior work has left unanswered: How do subsurface crack morphology and damage mode evolve with increasing contact load and number of reciprocating passes in each of these three material systems? At what load and cycle thresholds do transitions occur between deformation-dominated and fracture-dominated wear regimes? And how do microstructural features, namely strengthening precipitates, brittle oxide coatings, and ceramic particle reinforcements, govern the type, depth, and progression of subsurface cracking under Hertzian, scratch, and nanoindentation contact conditions? By answering these questions through multi-scale, cross-sectional characterization, this work establishes mechanistic damage maps that link intrinsic material architecture to contact damage resistance, providing a scientific basis for informed material selection and surface engineering design.

This study aims to investigate the mechanisms governing crack initiation, propagation, and subsurface damage in AA7075-T6 alloy, Anodized AA6061-T6 alloy, and Al-15 vol. 15%SiC composite under controlled scratching and indentation loading. By employing scratching, Hertzian indentation and nanoindentation techniques, the study seeks to characterize the mechanical response, deformation behavior, and damage evolution of these materials under localized contact stresses. Emphasis is placed on identifying the possible onset and progression of cracking, as well as the associated subsurface deformation mechanisms that develop during loading.

Likewise, the study targets establishing correlations between the observed surface and subsurface failure modes and the intrinsic material properties, including hardness (H), elastic modulus (E), and microstructural characteristics. Through a comparative analysis of the three material systems, the work also evaluates how variations in alloy composition, the presence of an anodized surface layer, and ceramic reinforcement influence crack formation, deformation mechanisms, and resistance to damage under contact loading. Ultimately, this investigation examines the wear and indentation behavior of Al-based materials and contributes to a deeper understanding of their structural integrity and performance under mechanical contact conditions.

## 2. Materials

**Figure 1:**
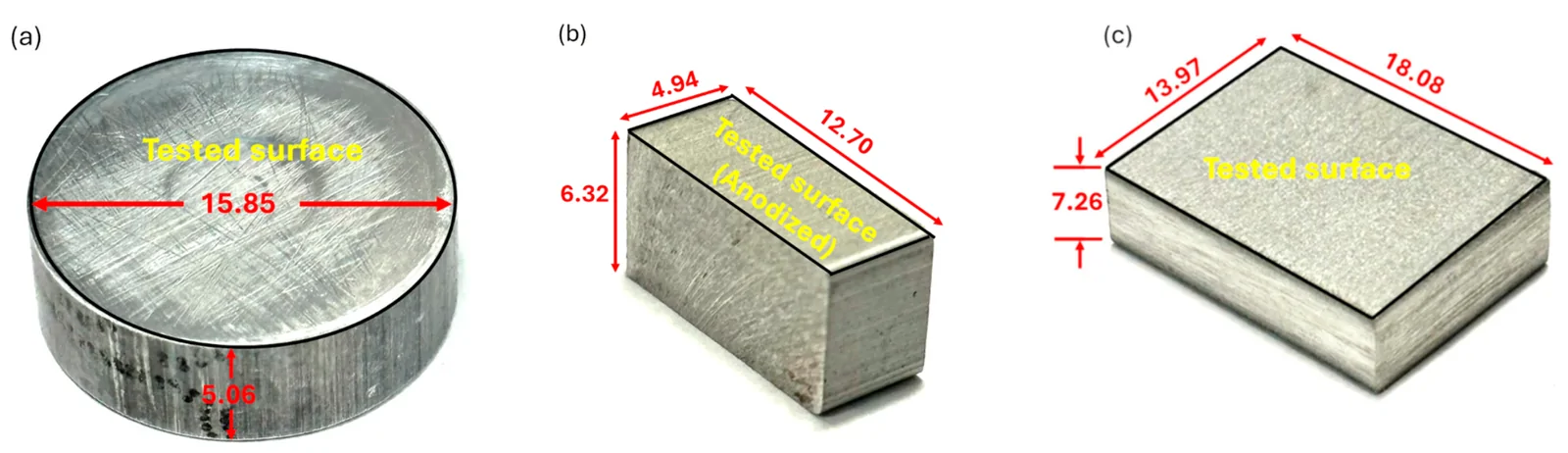
Tested Specimens with dimensions (mm); (**a**) AA7075-T6; (**b**) Anodized AA6061-T6; (**c**) Al-15 vol.% SiC T4 composite.

The materials selected for this investigation; AA7075-T6, anodized Anodized AA6061-T6, and Al-15 vol.% SiC T4 represent three distinct classes of Al based systems commonly used in structural and tribological applications, each exhibiting unique mechanical and damage-evolution characteristics. As shown in Figure 1, all specimens were prepared to the specified dimensions in accordance with processing parameters and testing conditions. The distinctive specimen geometries resulted from the as-procured forms and dimensions of the three materials; however, the designated testing surfaces were prepared under consistent surface-treatment conditions and provided sufficient surface area and thickness to ensure reliable mechanical and tribological characterization. AA7075-T6 (Figure 1a) is a high-strength, precipitation-hardened Al alloy widely applied in structures susceptible to localized damage under contact loading making it a candidate for examining scratch and indentation induced crack initiation and subsurface deformation.

Anodized Anodized AA6061-T6 (Figure 1b) provides a contrasting case where a thermally stable and hard oxide layer is grown on the alloy surface to enhance H, reduce wear, and improve corrosion resistance; this oxide layer significantly alters the stress distribution during scratching and influences the formation and evolution of microcracks at the coating-substrate interface. The Al-15 vol.% SiC T4 (Figure 1c) composite material incorporates SiC particles to increase stiffness, H, and wear resistance, introducing particle-matrix interactions that strongly affect both surface fracture behavior and subsurface crack propagation. Studying these three materials together enables a comprehensive assessment of how alloy composition, surface modification, and particle reinforcement interact to govern crack evolution mechanisms under controlled scratch loading.

**Figure 2:**
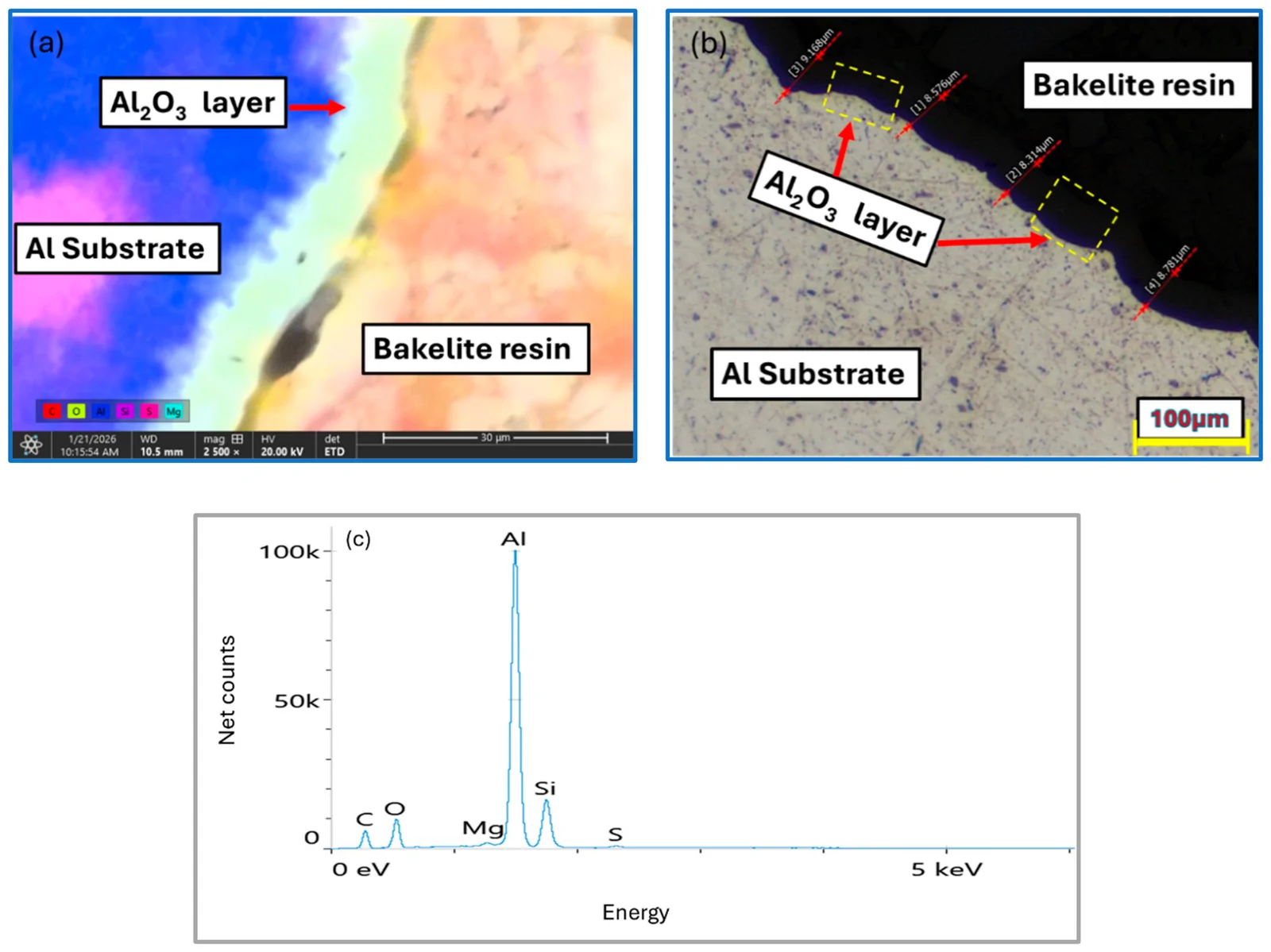
Cross-sectional mapping and thickness of oxide layer of Anodized AA6061-T6: (**a**) EDS; (**b**) CLSM; (**c**) Quant Map data.

Figure 2a,b illustrates the substrate-oxide layer interface in the Anodized AA6061-T6, revealing a relatively uniform oxide layer with an average thickness of 8.71 µm (Figure 2b). The well-defined interface suggests consistent oxide formation across the examined region. The energy-dispersive spectroscopy (EDS) analysis conducted at the interface, shown in Figure 2c, aligns with expectations, confirming the compositional characteristics of the oxide layer. The EDS spectrum indicates that Al, Si, and O are the predominant elements present, with their relative abundances decreasing in the order Al > Si > O, thereby validating the formation of an Al-Si oxide-rich layer at the interface.

The Al_2_O_3_ layer and the corresponding elemental composition shown were representative of the anodized surface, as consistent oxide-layer morphology and Al-O-Si elemental signatures were observed across multiple examined regions of the specimen.

### 2.1. Elemental Composition and Hardness

The bulk elemental compositions of the investigated materials were determined using inductively coupled plasma-optical emission spectroscopy (ICP-OES). Hardness measurements were performed following the Rockwell B scale in accordance with standards [12,13]. Sample surfaces were carefully prepared to be smooth, flat, and free of contaminants. Each specimen was mounted on a Rockwell hardness tester (Instron Wilson Hardness^®^, Rockwell 2000, Lake Bluff, IL, USA), and a 1/16-inch steel ball indenter was applied with a 10 kgf minor load followed by a 100 kgf major load. The instrument automatically recorded the indentation depth after a specified dwell time to determine the H. Measurements were taken at five well-spaced points on each specimen, and the average value was reported to ensure accuracy and reproducibility. ICP-OES analysis (Table 1) confirmed that the elemental compositions of the materials are consistent with their nominal alloy designations [14,15,16]. The measured H values (Table 2) further highlight the combined effects of alloy chemistry, reinforcement, and surface treatment on the mechanical response.

AA7075-T6 exhibited a relatively low Al content (90 wt.%), reflecting its highly alloyed nature. The presence of substantial Zn, Mg, and Cu contents is characteristic of high-strength 7xxx-series alloys [17] and is directly associated with pronounced precipitation hardening. These alloying additions are expected to significantly enhance yield strength (YS) and H. It demonstrated the highest average H (85.39 HRB), reflecting the chemistry and the associated hardening response. This elevated H is indicative of superior load-bearing capacity and resistance to plastic deformation.

AA6061-T6, primarily composed of Al with Mg and Si as the main alloying elements, also contains minor amounts of Fe and Cu. The Mg-Si system promotes the precipitation of magnesium silicide (Mg_2_Si), providing moderate strengthening while retaining inherent ductility. This balanced chemistry supports stable oxide film formation, contributing to moderate wear resistance under sliding conditions. Relative to the obtained EDX analysis in Figure 2 above, the elemental composition from ICP-OES (Table 1) represents the bulk composition of the specimens because the analysis was performed on the entire specimen rather than exclusively at substrate-anodized surface interfaces. Oxygen was therefore not included in the reported bulk composition as it constituted only a minor elemental fraction. In contrast, the EDX analysis in Figure 2 was performed locally at the substrate-anodized surface interface and is consequently more sensitive to the oxygen-rich surface oxide/anodized layer. The difference in oxygen content between the two analyses therefore reflects the different sampling regions and analytical techniques rather than a lack of repeatability. Accordingly, the ICP-OES and EDX results are considered complementary, with ICP-OES representing the bulk alloy chemistry and EDX representing the localized surface chemistry. Although the AA6061 substrate exhibited the lowest bulk H (50.18 HRB), anodization significantly increased the surface H (55.80 HRB), forming a hard oxide layer. This surface modification is expected to enhance durability and reduce material removal during sliding contact, improving overall tribological performance despite the relatively soft substrate.

The Al-15 vol.% SiC T4 composite showed a reduced Al fraction, attributable to the incorporation of reinforcement and secondary phases. Elevated concentrations of Cu, Mg, and Si were detected, which contribute to matrix strengthening. In combination with the SiC reinforcement, this compositional profile is expected to result in superior hardness, load-bearing capacity, and wear resistance compared to monolithic Al alloys. The presence of hard ceramic phases is particularly beneficial in mitigating material removal during sliding, thereby enhancing the overall tribological performance. The high average H (81.84 HRB) observed is attributable to the presence of hard ceramic SiC reinforcement and matrix strengthening from secondary alloying elements.

### 2.2. Microstructural Examination

To reveal the microstructure and surface morphology of the test specimens, standard metallographic preparation procedures were employed [18]. The specimens were initially subjected to successive grinding using progressively finer abrasive media, followed by mechanical polishing to obtain a mirror-like surface free from deformation and surface artifacts. Subsequently, the polished specimens were etched to enhance microstructural contrast. Etching was performed using Keller’s reagent [19], composed of 95 mL distilled water, 2.5 mL HNO_3_, 1.5 mL HCl, and 1 mL HF. Each specimen was immersed in the etchant for approximately 10–30 s, depending on the material response, to adequately delineate grain boundaries and constituent phases. Following etching, the specimens were immediately rinsed under running distilled water, then sprayed with ethanol to displace residual water and minimize staining. The samples were finally air-dried and visualized under confocal laser scanning microscope (CLSM) (Keyence VK-X1000, Chicago, IL, USA). The average surface roughness of each specimen prior to mechanical testing was also determined from CLSM.

X-ray diffraction (XRD) analysis was performed to characterize the crystal structure and identify the constituent phases of the materials investigated. Measurements were carried out using a Bruker D8 Advance X-ray diffractometer (Bruker, Billerica, MA, USA) equipped with a high-speed LynxEye™ detector and a copper X-ray source. The instrument was operated at an accelerating voltage of 40 kV and a current of 40 mA, employing Cu Kα radiation with a wavelength of 1.5418 Å. Diffraction data were collected over a 2θ range of 20–100° using a step size of 0.050°. The acquired diffraction patterns were analyzed using Bruker DIFFRAC.EVA software (DIFFRAC.EVA V7, DIFFRAC.Part 11 V8) and phase identification was achieved by comparison with reference patterns from the International Centre for Diffraction Data (ICDD) Powder Diffraction File (PDF) database.

## 3. Experimental

### 3.1. Nanoindentation Testing

Nanoindentation testing was carried out on the specimens to evaluate their near-surface mechanical response. Prior to testing, each specimen was mounted in Bakelite mounts approximately 1 inch in height to provide adequate support and stability during testing. The mounted samples were then subjected to a standard metallographic preparation procedure involving sequential grinding and polishing down to 0.5 µm for a highly smooth surface suitable for nanoindentation measurements. The final surfaces were left unetched to avoid introducing surface relief or chemical alterations that could influence the indentation response.

**Figure 3:**
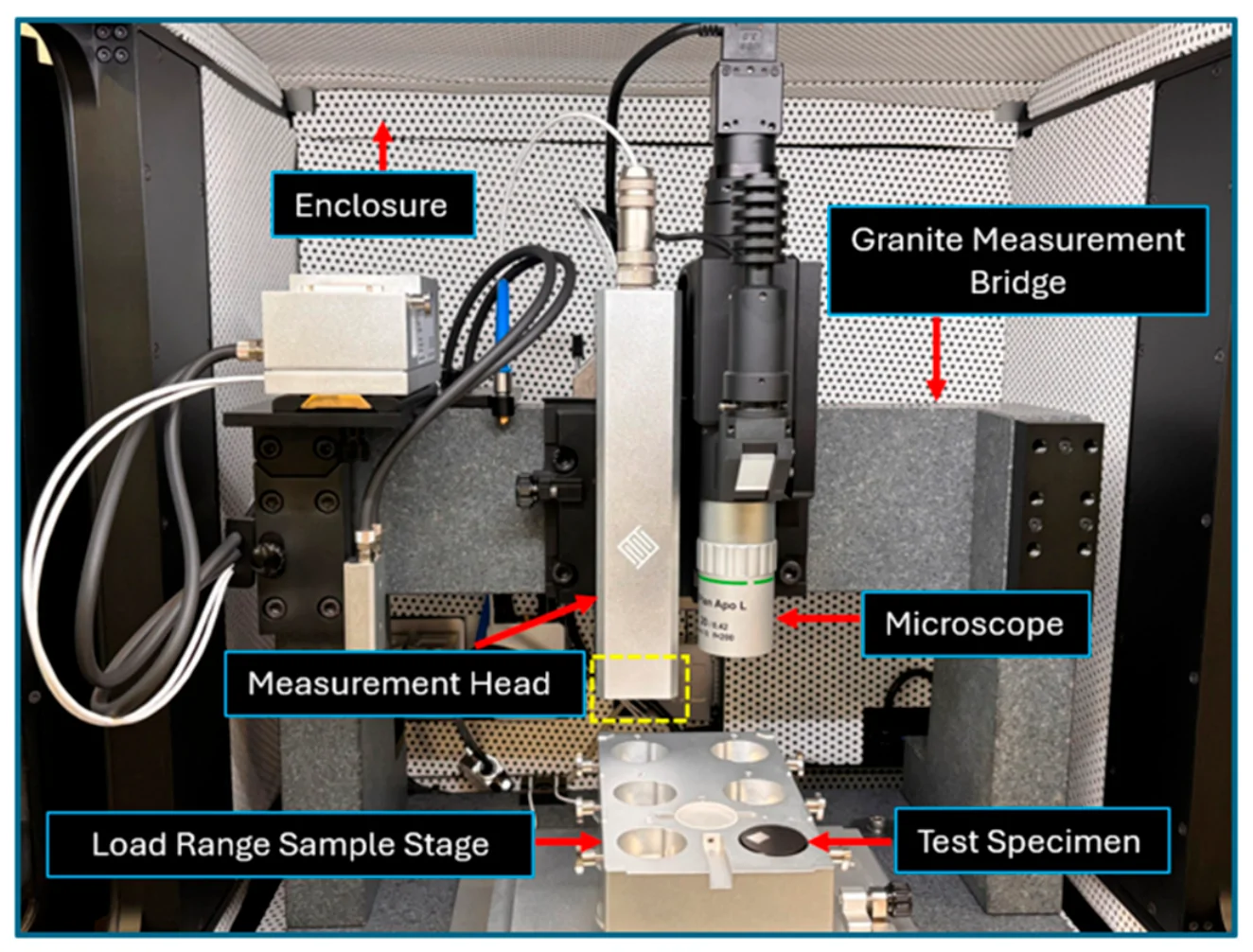
FT-104 nanoindentation testing system and identification of the principal components within the testing chamber.

The tests were performed utilizing FT-104 Nanoindenter with diamond Berkovich indenter (Femto Tools AG, Buchs/ZH, Switzerland) shown in Figure 3. The nanoindentation system was equipped with an integrated optical microscope, which was used to visually locate and select appropriate regions on the specimen surface prior to indentation. The microscope also enabled accurate positioning of the indenter tip and ensured that indentations were placed on defect-free areas while maintaining the required spacing between individual indents. The equipment was operated in displacement-controlled mode to ensure precise control of the indenter penetration during the loading cycle. During the approach stage, the indenter tip advanced toward the specimen surface with an approach step of 12.5 µm and a slow approach speed of 0.02 µm/s to accurately detect the surface and minimize impact effects. A trigger force window between -20 µN and 20 µN was applied to detect the point of initial contact between the indenter and the specimen surface. Signal noise was minimized using a filter frequency of 400 Hz. The indentation locations were separated by a lateral distance of ~120–150 µm to avoid interaction between neighboring plastic zones or residual stresses generated by adjacent indents.

For the indentation cycle, a maximum force of 200,000 µN was applied, while the maximum penetration depth was limited to 1.5 µm. Loading was performed at a rate corresponding to 0.05 µm/s, allowing the indenter to gradually penetrate the material while maintaining stable control of the displacement. The loading ramp time was set to 30 s to ensure smooth application of the load. Once the peak load was reached, the indenter was held at the maximum load for 5 s to allow time-dependent deformation mechanisms such as creep or viscoelastic relaxation to stabilize. Only one hold segment was used during the loading stage. Unloading was then carried out rapidly at a rate of 50 µm/s, with an unload ramp time of 0.03 s, allowing recovery of the elastic component of the deformation. The test concluded at an end force of 400 µN with no additional end hold time. Throughout the indentation cycle, data were collected at a sampling rate of 100 Hz to ensure accurate acquisition of load–displacement data for subsequent mechanical analysis.

In addition to the quasi-static indentation measurements, Continuous Stiffness Measurement (CSM) was implemented during the tests to monitor the dynamic contact stiffness as a function of indentation depth. The CSM module was operated in depth-controlled mode with a harmonic oscillation frequency of 75 Hz and a displacement amplitude of 0.002 µm. An amplitude ratio of 0% was maintained during the measurements to ensure stable oscillatory loading conditions. The incorporation of the CSM technique enabled continuous evaluation of stiffness and mechanical properties throughout the indentation process, thereby providing advanced description of the depth-dependent mechanical behavior of the tested materials.

The Berkovich tip area function was calibrated using a polished fused-quartz reference standard. The frame-compliance correction established during instrument commissioning was applied to the measurements, and no thermal-drift correction was used. The tests were performed in displacement-controlled mode to a programmed maximum displacement of 1.5 µm.

**Figure 4:**
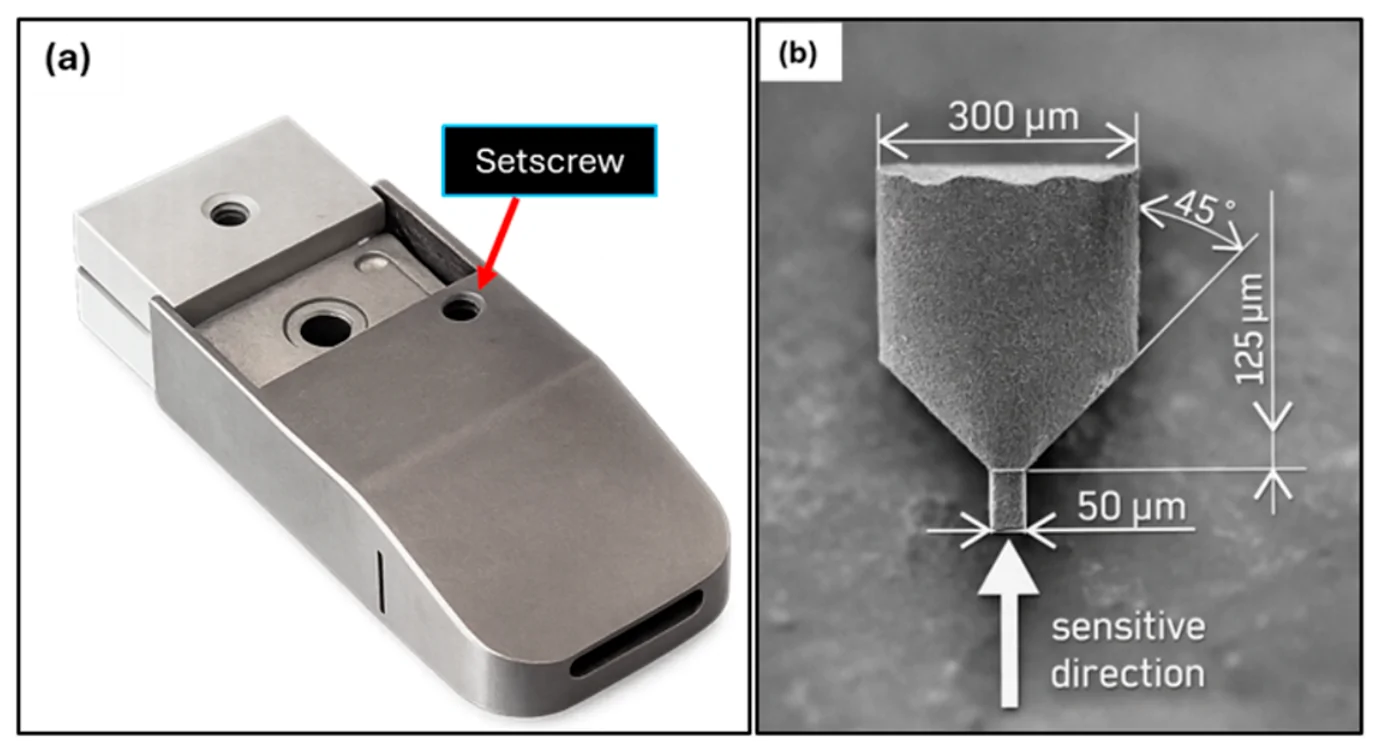
(**a**) Diamond Berkovich tip Microforce sensing probe; (**b**) Standard sensing probe tip geometry.

The measurement head of the nanoindentation system (yellow boxed region in Figure 3) houses the microforce sensing probe (Figure 4a), which serves as the primary component responsible for applying and measuring the indentation load during testing. This probe is equipped with a standard sensing probe tip, the geometry of which is illustrated in Figure 4b. The probe tip is designed to ensure precise contact with the specimen surface, enabling accurate measurement of the mechanical response during indentation. The diamond Berkovich indenter used had a nominal tip radius of approximately 50 nm. The Berkovich geometry, characterized by a three-sided pyramidal shape, is specifically suited for nanoscale mechanical testing due to its ability to achieve a significantly sharper apex compared to the conventional four-sided Vickers indenter. The indenter possesses a total included angle of 142.3°, ensuring a well-defined contact geometry for accurate determination of hardness and reduced modulus [20].

Force measurement within the system is achieved through a microelectromechanical systems MEMS-based capacitive sensing mechanism. This MEMS capacitive sensor allows for highly sensitive detection of very small forces and displacements, which is essential for nanoindentation experiments where the applied loads and resulting penetrations occur at the micro- to nanoscale. The capacitive sensing technology provides high resolution and stability, enabling precise monitoring of the applied force and indentation depth throughout the loading and unloading cycles of the test.

Nanoindentation testing provided continuous measurements of load, contact depth, displacement, H, and corresponding reduced modulus (E_r_) at each CSM location throughout an indentation cycle. The H and E_r_ were respectively obtained directly by the nanoindentation system leveraging the principles of Oliver and Pharr’s definition of indentation H (Equation (1)) and the canonical Sneddon stiffness relations (Equation (2)) [21,22], which relate the contact stiffness (S) to the projected contact area (Ac) and elastic response. The measured Er was subsequently converted to the instantaneous elastic modulus (E) using the relationship established by Warren C. Oliver and George M. Pharr through the Oliver-Pharr method [23], as presented in Equation (3). This approach determines the projected indentation Ac from the unloading stiffness derived from the load–displacement response, enabling accurate evaluation of the E from the experimentally measured *E_r_*.

(1) $$H=\frac{P_{max}}{A_{c}}=\frac{P_{max}}{f(h_{c})}$$

where H is the instantaneous hardness, *P_max_* is the maximum load, A_C_ is the contact area, and h_c_ contact depth at P_max_.

(2) $$E_{r}=\frac{S}{2\beta }\sqrt{\frac{\pi }{A_{c}}}$$

where *E_r_* is the reduced modulus of material, S is the contact stiffness, and β is a correction factor.

(3) $$\frac{1}{E_{r}}=\frac{1-{vs}^{2}}{E_{s}}+\frac{{1-vi}^{2}}{E_{i}}$$

where *E_s_* is the Elastic modulus of material, *E_i_* is the Elasticity of indenter tip (1140 GPa), ***v***_S_ is the Poisson’s ratio of specimens (0.33), and ***v***_i_ Poisson’s ratio for diamond indenter (0.07).

### 3.2. Scratch Testing

**Figure 5:**
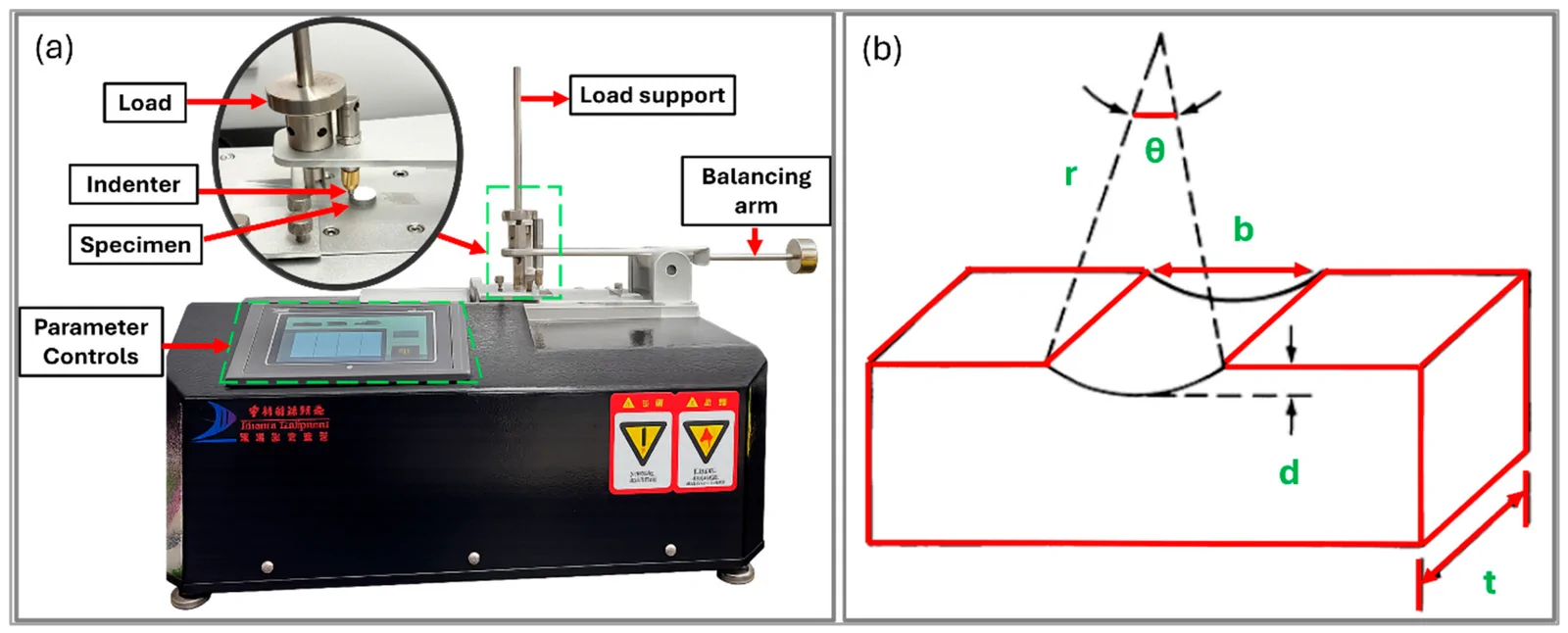
(**a**) Scratch Tester; Load-scratching mechanism (Insert); (**b**) Schematic representation of worn volume of block specimen.

Scratch testing was conducted to evaluate the surface and subsurface damage behavior of the specimens under controlled sliding contact conditions. Prior to reciprocating scratch testing, all specimens were prepared using the same procedures employed for metallographic sample preparation as described in Section 2.2 above. This surface preparation was carried out to eliminate processing-induced scratches, surface debris, and residual contaminants, thereby ensuring a uniform and reproducible surface condition for scratch testing. All tests were performed at room temperature using an automatic scratch tester shown in Figure 5a below (DZ-113, Zonhow Test Equipment Co., LTD, Dongguan, Guangdong, China). A spherical tungsten carbide (WC) indenter with a diameter of 1/8 inch was employed for all experiments. Scratches were produced over a fixed scratch length of 5 mm at a constant scratch speed of 1 mm/s.

For each test condition, a constant normal load (50 g, 100 g, 500 g, and 1000 g) was applied and maintained throughout the scratch. The normal load was applied prior to the initiation of scratching, and no-load ramping was employed during the test. Scratching was conducted in reciprocating mode, with the number of reciprocating passes set to 1, 4, 20, and 50 cycles per load. All tests were performed under identical environmental and operational conditions to ensure repeatability and comparability between test cases.

For multi-pass scratching, geometric relationships (Figure 5b, Equations (4–6)) based on the idealized scratch scar morphology were applied to estimate wear-related parameters.

(4) $$b=\mathrm{D sin}\frac{\theta }{2}$$

(5) $$V=\frac{D^{2}t}{8}(\theta -\sin\theta )$$

(6) $${\theta =2\sin}^{-1}\frac{b}{D}$$

where b is the average scratch width, D is the diameter of the indenter, V is scar volume, t is the block width, and θ is the sector angle.

Figure 5b represents the idealized cross-sectional geometry used to calculate the scratch scar volume from the measured width and depth. The pile-up and debris observed in the SEM analysis are used for qualitative interpretation of the wear mechanism and are not included in the geometric calculation. The calculated values are therefore reported as comparative geometric wear-volume estimates under the same test conditions.

Subsequently, specific wear rate, W_S_ (Equation (7)) [24] and scratch hardness, H_S_ (Equation (8)) [25] for a spherical indenter were determined. Collectively, these calculated parameters provided a quantitative basis for correlating contact mechanics with the experimentally observed surface and subsurface damage evolution.

(7) $$W_{s}=\frac{V}{F\times L}$$

where W_S_ is the specific wear rate, V is the volume loss, F and L are the load and total sliding distance respectively.

(8) $$H_{s}=\frac{8F}{\pi b^{2}}$$

where H_S_ is the scratch hardness.

The Hs is an indirect measurement of material’s resistance to plastic deformation during sliding contact and is commonly related to the applied load and the resulting groove width or penetration depth by the indenter. In reciprocating scratch testing, higher H_S_ generally indicates greater resistance to material removal, ploughing, and permanent surface deformation. The calculated H_S_ values for the three grades of Al varies with loads and number of passes attributable to the competing effects of strain hardening, elastic recovery, surface pile-up, oxide layer disruption, and localized softening during repeated sliding.

Following scratch testing, the resulting scratch tracks were characterized using CLSM and SEM. Quantitative and qualitative assessments were carried out to determine the variation of W_S_ and H_S_ with loads and passes, material pile-up, and subsurface cracking behavior. The detailed microstructural and morphological outcomes of the scratch tests are presented and discussed in the subsequent sections.

Generative AI disclosure:ChatGPT was used to assist in generating the schematic illustration of the wear and indentation mechanisms presented in the discussion section. The output was reviewed, modified, and scientifically validated by the authors.

### 3.3. Hertzian Indentation Testing

**Figure 6:**
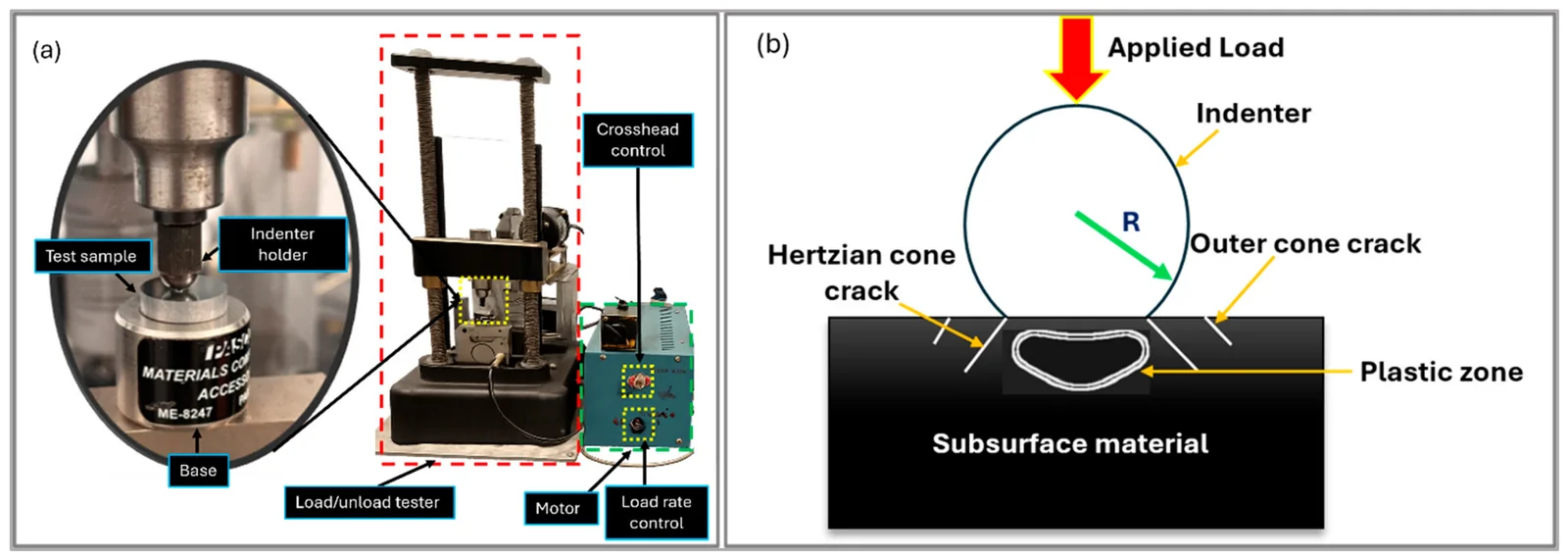
(**a**) PASCO ME-8236 Miniature Materials Testing System; (**b**) Schematic representation of indentation mechanism.

Hertzian indentation testing was also conducted on the three materials using a ME-8236 Miniature Materials Testing System (PASCO, Roseville, CA, USA shown in Figure 6a) to evaluate contact-induced deformation and possible crack initiation behavior under spherical loading conditions representative of Hertzian contact. Figure 6b indicates the loading and possible resulting deformation and cracking stemming from Hertzian contact. A 1/16-inch (1.59 mm) WC-6Co spherical indenter was employed to ensure elastic-plastic contact with high H contrast relative to the test substrates. Each specimen was subjected to a maximum applied load of 800 N, with a controlled loading rate of 0.1 mm/min to minimize dynamic effects and ensure quasi-static contact conditions. Upon reaching the peak load, a dwell time of 10 s was maintained to allow for stress stabilization and time-dependent deformation effects prior to unloading.

Following indentation, both surface and cross-sectional analyses were performed to characterize indentation morphology, possible crack patterns, and subsurface damage evolution. Surface topography and crack features were examined to obtain profiles of the residual impressions, including measurements of indent diameter, depth, and crack distribution where present.

For subsurface characterization, specimens were carefully sectioned through the center of the residual indent using the Buehler Isomet^®^ 1000 precision saw (Opti-Tech Scientific Inc., Whitby, ON, Canada) at a load of 350 N and a 400-rpm speed. The sectioned surfaces of samples were subsequently ground and polished following established metallographic preparation protocols to eliminate preparation-induced artifacts. Cross-sectional observations were then carried out using CLSM for depth profiling and SEM for high-resolution imaging of deformation zones, crack propagation paths, particle-matrix interactions (in the Al-15 vol.% SiC T4 composite), and coating-substrate interfacial behavior (in the Anodized AA6061-T6).

This combined surface and subsurface analytical approach enabled comprehensive assessment of Hertzian contact damage mechanisms, including elastic-plastic deformation, radial and lateral cracking, coating fracture, and reinforcement-induced stress concentration effects across the three material systems.

## 4. Results and Discussion

This section presents and discusses the experimental findings obtained for the AA7075-T6, Anodized AA6061-T6, and Al-15 vol.% SiC T4 Composite, with emphasis on their microstructural characteristics, phase constitution, mechanical properties, and tribological performance. The results are interpreted in relation to the processing condition and the resulting differences in microstructure and mechanical response. Particular attention is given to correlating the nanoindentation, scratch, and hertzian-type to elucidate the mechanisms governing the mechanical and wear behavior of the two materials.

The nanoindentation measurements were conducted at multiple spatially separated locations to account for local variations in the mechanical response. Nevertheless, independent duplicate datasets were not acquired for the measurements presented. Consequently, the H, E, W_S_, and H_S_ values reported herein are interpreted as comparative responses under the specified experimental conditions rather than statistically replicated measurements. Accordingly, no claims of statistical repeatability or experimental scatter are made from these datasets. Independent replicate testing in future work would be required to quantitatively establish the repeatability and statistical variability of these properties.

### 4.1. Microstructural Characterization

**Figure 7:**
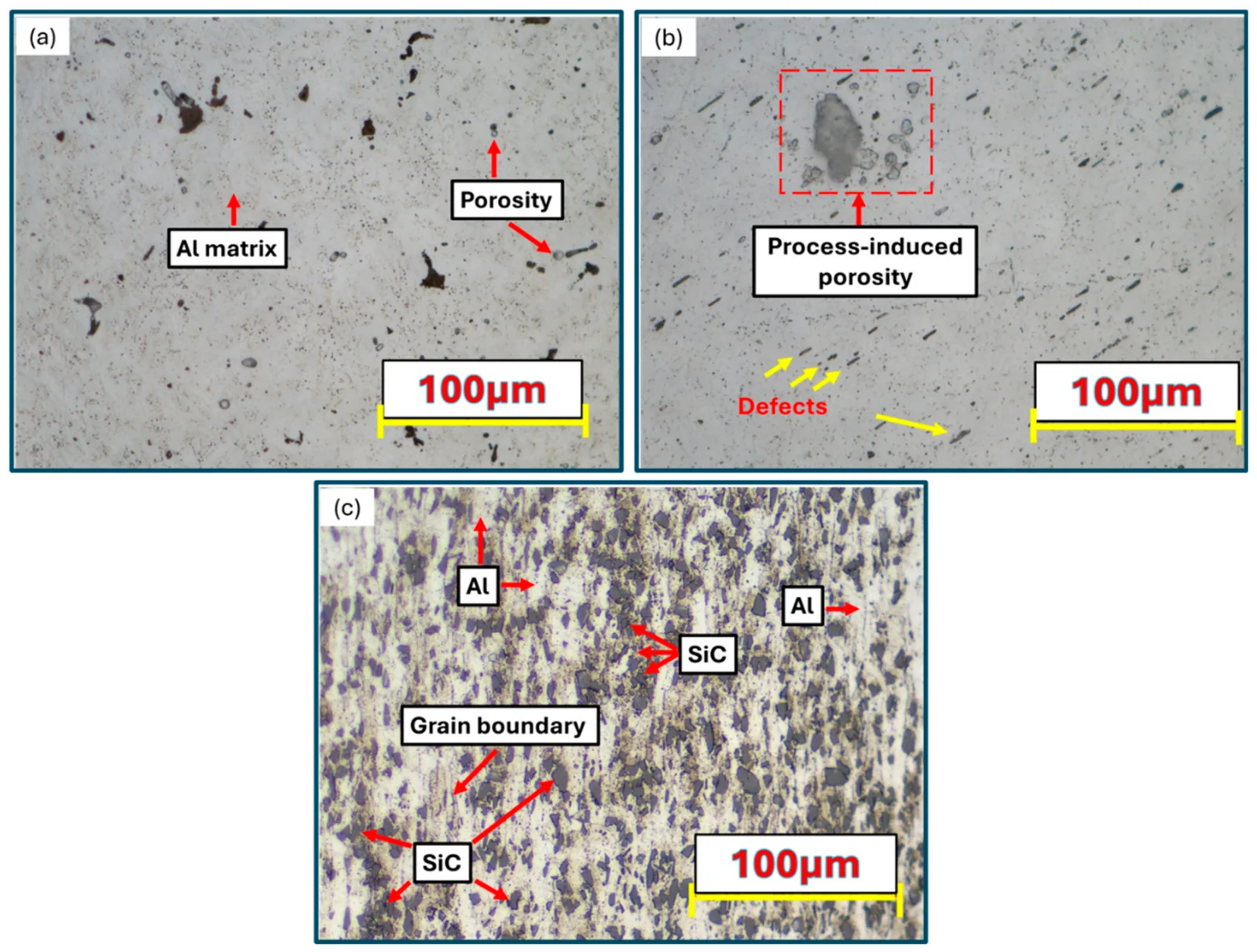
Micrographs (100×) of etched specimens; (**a**) AA7075-T6; (**b**) AA 6061 (Anodized surface); (**c**) Al-15 vol.% SiC T4 composite.

The etched specimens exhibited distinct surface morphologies, as evidenced by their respective metallographic profiles (Figure 7). These variations in topography are expected to significantly influence their mechanical response under scratch and indentation loading. Accordingly, the combined effects of surface roughness (Ra) and intrinsic microstructural characteristics play a critical role in governing the scratch and indentation wear behavior of these alloys.

The etched AA7075-T6 surface (Figure 7a) predominantly revealed the α-Al matrix with localized porosity. These pores likely arise from micro-segregation and the selective dissolution of secondary intermetallic phases during etching. Such localized attack introduces micro-pits and surface discontinuities, resulting in a relatively elevated average Ra of 4.15 µm. From scratch and indentation perspective, this high roughness implies increased asperity interaction during initial contact. Under scratch loading, pronounced surface peaks can intensify localized stress concentrations, promoting early plastic deformation and micro-cutting. Similarly, during indentation, pre-existing surface irregularities may contribute to non-uniform load distribution and localized strain accumulation, potentially accelerating crack initiation at pore sites.

In contrast, the anodized surface of the AA 6061 specimen (Figure 7b) exhibited the Al_2_O_3_ matrix with porosities inherent to the anodization process and minor surface defects yet demonstrated a significantly lower average roughness of 1.77 µm. The comparatively smoother surface suggests a more uniform topography with reduced asperity amplitude. The finer and more homogeneously distributed Mg_2_Si precipitates in AA 6061 likely resulted in less aggressive preferential dissolution during etching, thereby minimizing surface height variations. Under scratch loading, a smoother surface generally promotes more stable ploughing with reduced penetration depth fluctuations. During indentation, improved surface uniformity supports more symmetric plastic flow beneath the indenter, potentially leading to more predictable deformation behavior and reduced stress concentration compared to the rougher alloys.

The Al-15 vol.% SiC T4 composite (particle size range of 3.1–8.3 µm) displayed a heterogeneous two-phase structure, consisting of a bright Al matrix and dark SiC reinforcement particles (Figure 7c). The composite exhibited the highest average roughness, 4.26 µm, attributable to the differential etching response between the relatively inert SiC particles and the more reactive Al matrix. This phase contrast introduces micro-scale height discontinuities at particle-matrix interfaces. From a wear standpoint, such heterogeneity significantly influences scratch response: the hard SiC particles can act as load-bearing constituents, resisting penetration and reducing matrix ploughing, but may also induce stress concentration at the interface. Additionally, particle pull-out or interfacial debonding under scratch loading could contribute to three-body abrasion, increasing material removal. During indentation, the presence of rigid ceramic reinforcements constrains plastic flow in the surrounding matrix, potentially enhancing H while promoting localized interfacial stresses.

Largely, the observed roughness values correlate strongly with microstructural heterogeneity and phase contrast. The smoother AA 6061 surface is expected to exhibit more stable scratch tracks and uniform indentation imprints, whereas the rougher AA7075-T6 and Al-15 vol.% SiC T4 surfaces may experience greater stress localization at asperities. However, in the case of the composite, the increased roughness is coupled with reinforcement-induced load sharing, which may offset the detrimental effects of surface irregularities by improving resistance to penetration and plastic deformation.

### 4.2. X-Ray Diffraction Analysis

**Figure 8:**
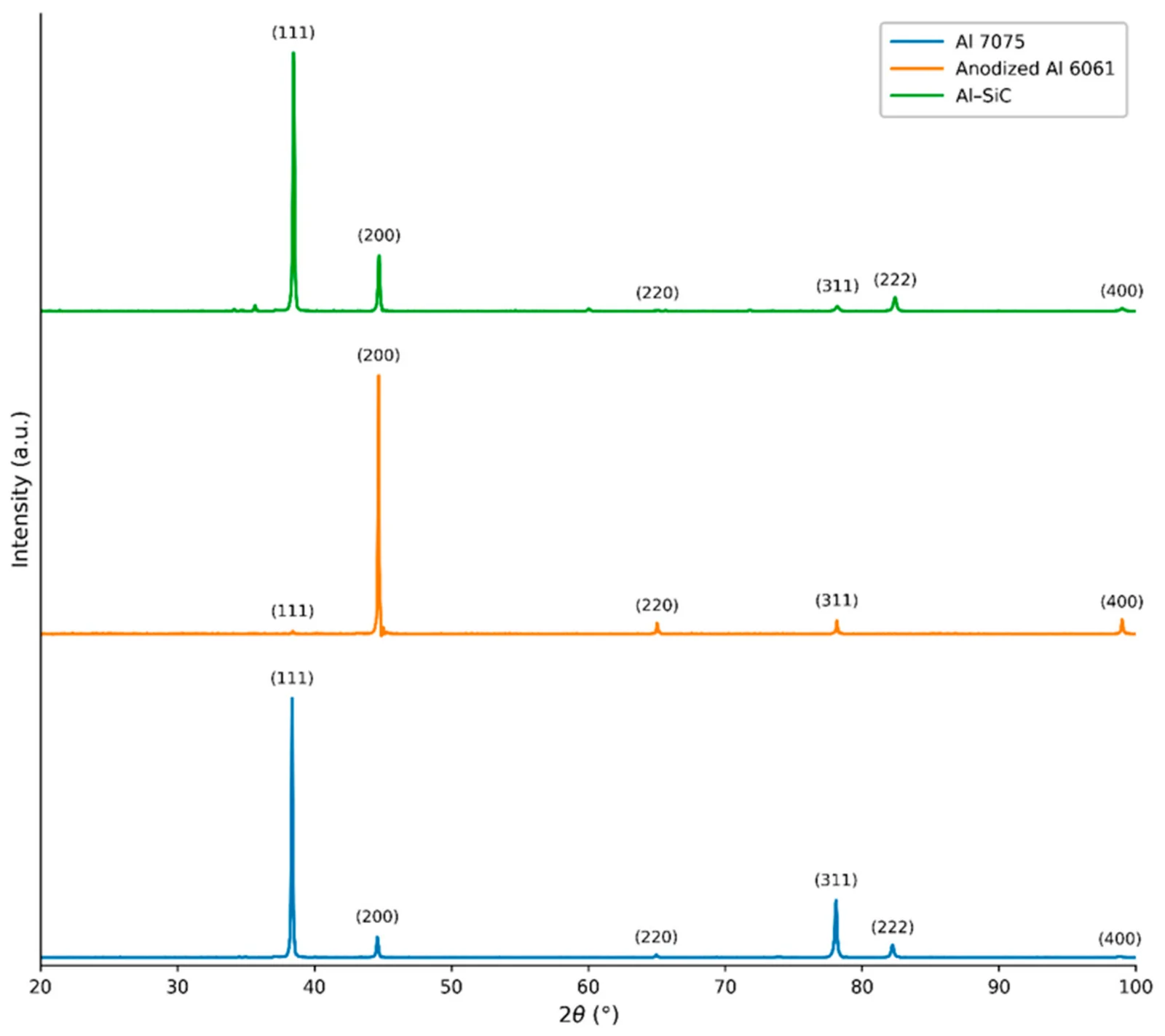
X-Ray diffraction patterns of all three materials.

The XRD patterns of the investigated materials were predominantly characterized by reflections corresponding to the FCC α-Al phase (Figure 8), indexed to the (111), (200), (220), (311), and (400) planes, consistent with standard ICDD references: 01-072-3440 (AA7075-T6), 04-026-4280 (Anodized AA6061-T6), and 04-003-7126 (Al-15 vol.% SiC T4 composite). However, notable differences were observed in the relative peak intensities, indicating variations in preferred crystallographic orientation (texture) and phase constitution. These differences reflect distinct microstructural evolution driven by alloy chemistry, reinforcement incorporation, and processing history. Such crystallographic variations may contribute to mechanical anisotropy and, consequently, influence the materials’ response under scratch and indentation loading.

For AA7075-T6, the (111) plane exhibited the highest intensity, indicating a dominant crystallographic orientation parallel to the surface. This preferential orientation may be attributed to thermomechanical processing and the influence of alloying elements such as Zn, Mg, and Cu, as identified by ICP-OES. The presence of strengthening precipitates (e.g., MgZn_2_ and Al_2_CuMg) may also contribute to localized lattice distortions, subtly influencing peak intensities while remaining difficult to resolve as distinct secondary peaks due to their fine dispersion.

In the case of AA 6061-T6, the (200) plane was dominant, suggesting a different texture evolution compared to AA7075-T6. The Mg and Si contents determined by ICP-OES support the presence of Mg_2_Si precipitates; however, their limited volume fraction and nanoscale distribution likely resulted in weak or overlapping reflections. The dominance of the (200) peak may reflect rolling- or extrusion-induced texture, as well as differences in strain energy minimization during processing.

For the Al-15 vol.% SiC T4 composite, the (111) plane showed the highest intensity, indicating yet another distinct preferential orientation. While the diffraction pattern was still largely governed by the α-Al matrix due to its higher volume fraction, the incorporation of SiC reinforcement can influence matrix solidification behavior and residual stress distribution, thereby affecting texture development. Although distinct SiC peaks may be present depending on reinforcement content and particle distribution, their relative intensity can be lower than that of the matrix, particularly if the Al phase dominates the sampled volume.

The XRD patterns were used for phase identification and qualitative comparison of the main FCC alpha-Al reflections. AA7075-T6 showed its strongest reflection at (111), Anodized AA6061-T6 at (200), and the Al-15 vol.% SiC T4 composite at (111). Differences in relative peak intensity suggest preferred orientation, but quantitative texture coefficients were not calculated because pole-figure and reference-normalized measurements were not available.

### 4.3. Nanoindentation

#### 4.3.1. Indented Surface Morphologies

**Figure 9:**
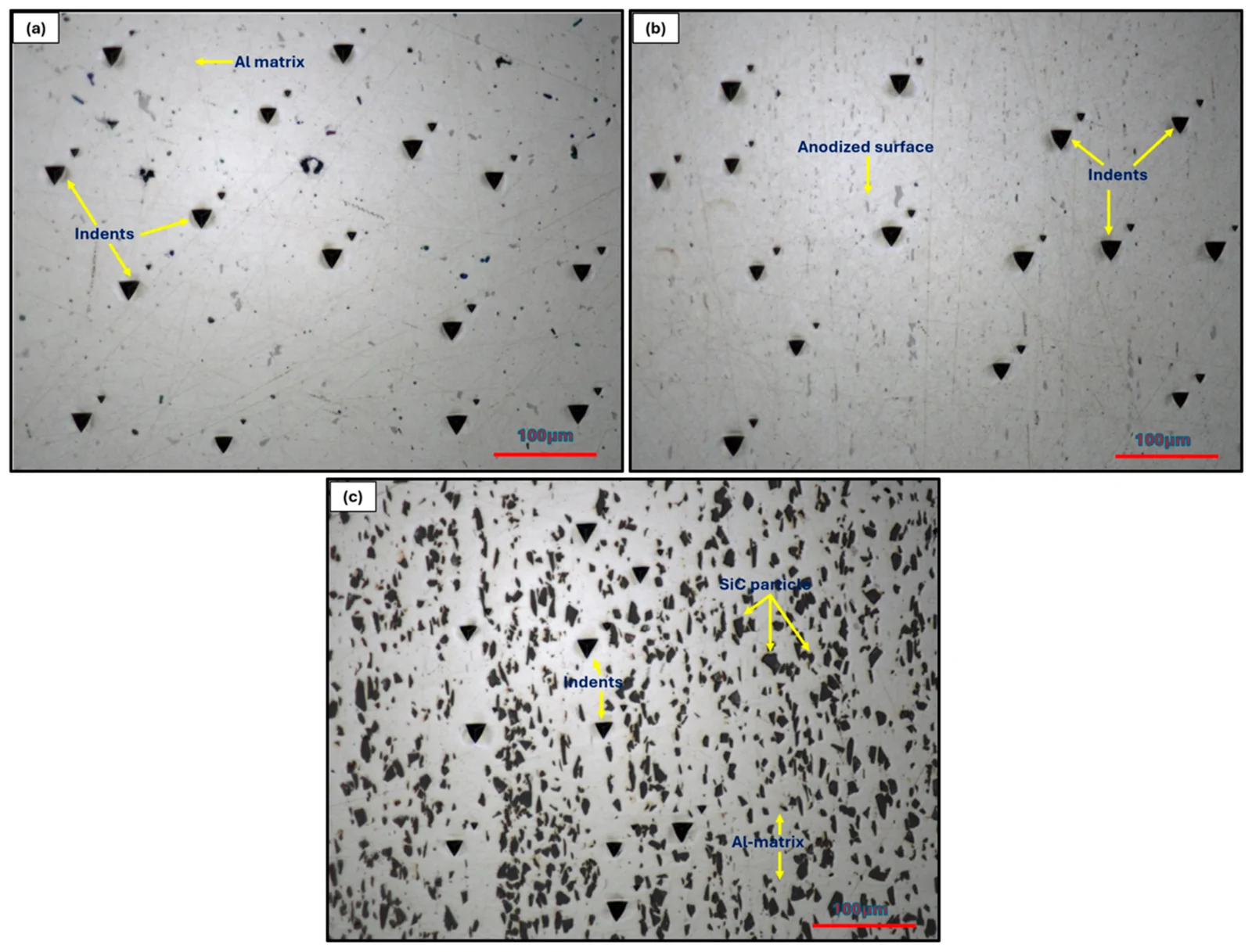
Nanoindented images of specimens’ surface: (**a**) AA7075-T6; (**b**) Anodized AA6061-T6; (**c**) Al-15 vol.% SiC T4.

The distribution of the nanoindentation marks in Figure 9 indicates that most indentations were performed within the Al-rich matrix. Therefore, the measured H and E response primarily reflects the local mechanical behavior of the matrix and should not be interpreted as intrinsic properties of the SiC reinforcement. The mechanical response of the Al matrix is governed by its metallurgical state, including the effects of alloying elements present in solid solution, precipitation or second-phase strengthening, and local microstructural heterogeneity. In the Al-15 vol.% SiC T4 composite, the SiC particles can additionally affect the matrix response through load transfer and constraint of plastic deformation, particularly when a particle or particle-matrix interface lies within or near the indentation deformation zone. Consequently, some point-to-point variation in the measured nanoindentation properties is expected because of the heterogeneous distribution of the reinforcement. Since elemental mapping was not conducted in the present study, the contribution of individual alloying elements or their local concentrations cannot be quantitatively separated; therefore, the nanoindentation results are discussed in terms of the combined effects of matrix composition, microstructure, and reinforcement rather than assigning the measured response to a specific alloying element.

The surface morphologies of the indented specimens (Figure 9) were observed using the embedded microscope in the FT-104 Nanoindentation testing system. The micrographs reveal the residual indentation impressions together with the surrounding microstructural features characteristic of each material. In the AA7075-T6 specimen (Figure 9a), the images show clearly defined indents within the Al matrix. For the Anodized AA6061-T6 specimen (Figure 9b), the surface is characterized by distinct indentation impressions within the anodized layer. In contrast, the Al-15 vol.% SiC T4 (Figure 9c) composite displays indentation marks within the Al matrix together with the presence of dispersed SiC reinforcement particles embedded in the matrix.

The variation in residual indentation sizes, with relatively large and small impressions occurring in close proximity, is not attributed to the absence of pre-indentation step, as surface contact was systematically established using the controlled approach and trigger-force procedure. Instead, the variation likely reflects local differences in mechanical response arising from microstructural heterogeneity, crystallographic orientation, elastic recovery, and differences in pile-up or sink-in behavior [26,27]. These indentation impressions represent the localized deformation produced during nanoindentation and served as the basis for extracting the mechanical response parameters. The relationships among these parameters explain the mechanical response of the materials under localized contact loading. A detailed analysis of these relationships and their implications for the mechanical behavior of the materials investigated is presented in the subsequent sections of this text.

#### 4.3.2. Load–Displacement and Contact Depth Analysis

**Figure 10:**
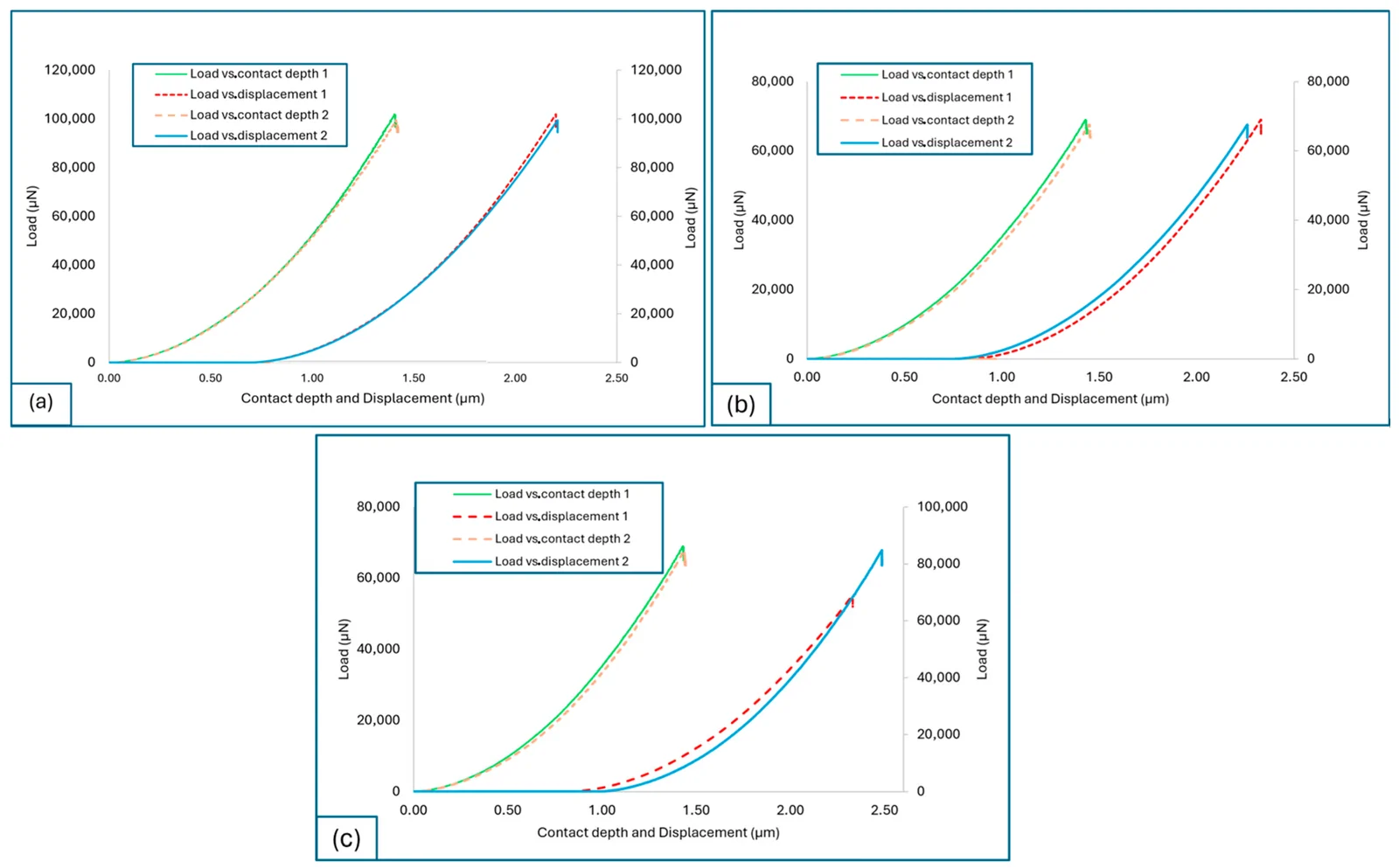
Load-depth/displacement curves; (**a**) AA7075-T6; (**b**) Anodized AA6061-T6; (**c**) Al-15 vol.% SiC T4.

The load-depth responses (Figure 10) present representative nanoindentation curves selected from approximately 30 indents performed on each material. The selected curves are representative of the overall response observed across the complete dataset and illustrate the localized deformation behavior under progressively increasing contact load. The duplicated curves exhibited generally consistent responses, although slight variations in maximum contact depth and displacement were observed between the two indentation points. For AA 7075-T6, the nearly identical curves indicate a relatively uniform mechanical response at the examined locations. The small variations observed for anodized AA6061-T6 may be associated with local variations in the anodized layer and its underlying substrate. In Al-15 vol.% SiC-T4, the observed larger displacement variation can reasonably be attributed to the heterogeneous microstructure, as the indentation locations may correspond to the Al matrix, SiC reinforcement, or the Al-SiC interfacial region. Largely, the duplicate curves demonstrate good reproducibility while also capturing the expected local microstructural variability of the examined materials. In all cases, the plots show the relationship between the applied load and both the contact depth and the total displacement during indentation. As mentioned above, the measurements were conducted in CSM mode, which enables the probe to oscillate during loading so that stiffness and unloading responses are measured continuously throughout the indentation cycle. Consequently, a long final unloading segment was not required. The small plateau observed near the peak load in the curves corresponds to the holding segment (5–10 s) introduced at the maximum load to allow partial stress relaxation at the probe-sample interface.

For the AA7075-T6 specimen (Figure 10a), the load-depth curves show a smooth nonlinear increase in both contact depth and total displacement with increasing applied load, reaching approximately 100,000 µN at the maximum indentation load. The displacement curve consistently lies to the right of the contact depth curve, reflecting the expected difference between the total penetration depth and the true contact depth after accounting for elastic recovery. The gradual curvature of the plots indicates a progressive increase in resistance to penetration as the indenter encounters the precipitation-strengthened Al matrix. The limited separation between the curves suggests relatively moderate elastic recovery compared with softer alloys, which is consistent with the relatively high strength and H typically associated with AA7075-T6.

For the Anodized AA6061-T6 specimen (Figure 10b), the load-depth relationship exhibits a similar nonlinear trend but reaches a lower peak load of approximately 70,000 µN. The curves again show the expected separation between contact depth and displacement, reflecting elastic recovery of the material during indentation. The presence of the anodized oxide layer contributes to an initially, slightly, higher resistance to penetration at shallow depths, while the underlying Al substrate accommodates further deformation as the load increases. This layered mechanical response contributes to the curvature observed in the plot.

For the Al-15 vol.% SiC T4 composite (Figure 10c), the load-depth response also reaches approximately 70,000 µN but shows a slightly steeper rise in load with respect to depth compared with the anodized Al specimen. This behavior reflects the reinforcing effect of the dispersed SiC particles, which locally constrain plastic deformation within the Al matrix and increase resistance to indenter penetration. The separation between the contact depth and displacement curves again reflects elastic recovery of the composite system after partial unloading at the contact interface. The reinforcement particles contribute to a more heterogeneous deformation response, where localized strengthening around the particles limits excessive penetration of the indenter.

#### 4.3.3. Elasticity and Hardness vs. Contact Depth

**Figure 11:**
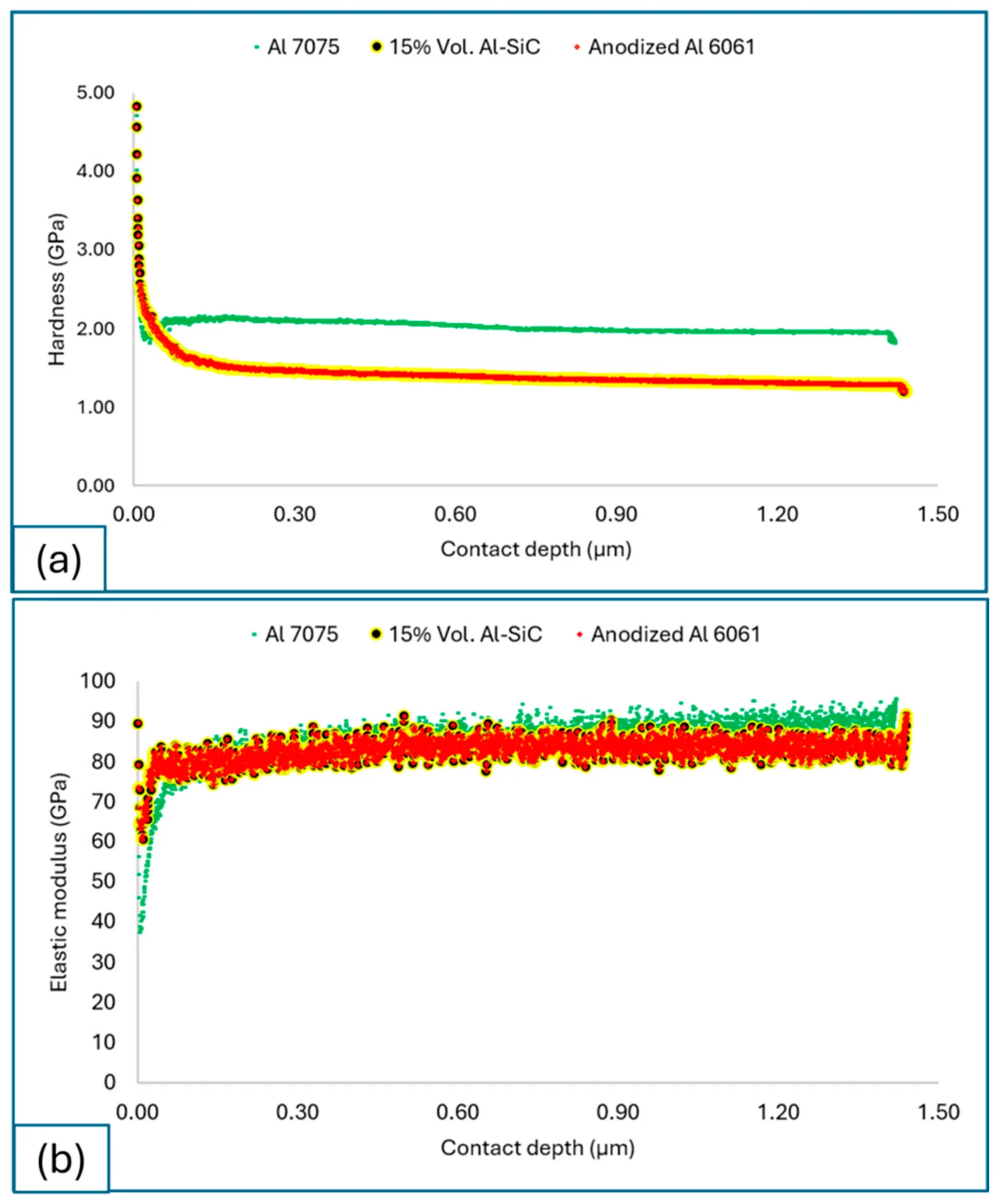
(**a**) Hardness and (**b**) Elastic modulus; vs. contact depth curves for materials.

The nanoindentation responses of the three materials show the characteristic depth-dependent mechanical behavior typically observed during CSM testing, where both H and E rapidly stabilize after the initial surface contact (Figure 11). The early fluctuations at very shallow depths arise from tip-surface interaction effects, surface roughness, oxide layers, and uncertainties in determining the true contact area. Once the indenter penetrates beyond the near-surface region, the measured properties converge toward values representative of the bulk or coating microstructure.

The 1.5 µm setting was the programmed displacement endpoint. The comparison in Figure 11 focuses on the more stable response between approximately 0.1 and 1.4 µm. Values below approximately 0.1 µm are shown but are not used for quantitative comparison because they are more sensitive to contact-point and contact-area uncertainty. The early scatter may also reflect surface roughness, local oxide or particle heterogeneity, and CSM signal noise.

For AA7075-T6 (Figure 11a), the H curve exhibits a strong indentation size effect at the smallest depths, initially exceeding 4.9 GPa before decreasing sharply and stabilizing near approximately 1.5–2.0 GPa at deeper penetration. This rapid decline is characteristic of nanoindentation measurements where the apparent H is artificially elevated due to geometrically necessary dislocations generated beneath the indenter at small indentation volumes. As the indentation volume increases, dislocation density becomes more representative of the bulk plastic response, leading to the observed stabilization. The relatively moderate H reflects the precipitation-strengthened microstructure of AA7075-T6, where strengthening arises primarily from η′ (MgZn_2_) precipitates and solid-solution contributions [28]. The stable E (Figure 11b) plateau indicates that the nanoindentation depth was sufficient to minimize surface artifacts and capture the intrinsic elastic response of the alloy matrix. The E initially fluctuates significantly at shallow contact depths below approximately 0.05 μm, reaching transient values near 100 GPa before rapidly stabilizing. Beyond roughly 0.1 μm, the modulus converges to a steady plateau between approximately 80–90 GPa, remaining relatively constant up to the maximum contact depth of about 1.4 μm. This value aligns well with reported E values for wrought AA7075-T6 alloys, typically around 71–80 GPa [14] depending on temper condition and microstructural anisotropy.

In the anodized AA6061 specimen, the H profile (Figure 11a) exhibits values exceeding 4.8 GPa at shallow penetration depths before decreasing rapidly and stabilizing at approximately 1.2–1.4 GPa with increasing indentation depth. This behavior reflects the depth-dependent mechanical response of the anodic Al_2_O_3_ layer and its interaction with the underlying Al substrate. At shallow indentation depths, the indenter primarily probes the hard ceramic oxide, resulting in high measured H due to the strong resistance of the anodic layer to plastic deformation. As the indentation depth increases, the measured H decreases because the plastic deformation zone beneath the indenter extends progressively through the porous anodic oxide toward the oxide/substrate interface. Consequently, the measured H increasingly reflects the effective mechanical response of the anodic coating and the more compliant Al substrate, even though the indenter itself may remain entirely within the oxide layer. This depth-dependent transition is characteristic of coated systems and arises from the combined effects of the indentation size effect, the evolving deformation and localized damage within the anodic oxide, and the increasing influence of the substrate as the deformation field extends deeper into the material. The E (Figure 11b) profile similarly stabilizes after the initial indentation stage but exhibits slightly lower steady values, averaging approximately 75–85 GPa across the majority of the indentation depth range. The initial spikes around ~90 GPa at depths below roughly 0.02 μm are attributable to the stiff anodic oxide layer combined with measurement uncertainty during first contact. As the indentation progresses, the indenter likely transitions from the porous anodic oxide into the underlying Al substrate, producing the observed stabilization.

The Al-15 vol.% SiC T4 composite (particle size range of 3.1–8.3 µm) the H profile (Figure 11a) shows a similar indentation size effect as observed in the other materials, with very high values at shallow depths exceeding 4.9 GPa before decreasing rapidly and stabilizing around approximately 1.2–1.6 GPa at larger depths. The early H spikes likely occur when the indenter contacts or partially penetrates SiC reinforcement particles, which possess substantially higher H than the Al matrix. As the indentation depth increases, the measured H becomes an averaged response reflecting both the ductile matrix deformation and the constraint imposed by surrounding ceramic particles. The relatively stable H plateau suggests effective load transfer and particle reinforcement, which is consistent with previously reported nanoindentation studies of particle-reinforced Al composites where local stiffness and hardness depend strongly on particle distribution and the matrix-particle interface. It displays a slightly higher and more stable E (Figure 11b) response compared with the other materials, with the E plateauing around 80–90 GPa beyond approximately 0.1 μm contact depth. The higher modulus reflects the reinforcing effect of the SiC particles dispersed within the Al matrix. SiC possesses a very high elastic modulus (~410 GPa), and even modest volume fractions can significantly increase the effective stiffness of the composite through load transfer mechanisms between the matrix and reinforcement particles.

### 4.4. Evaluation of Wear Behaviour

**Figure 12:**
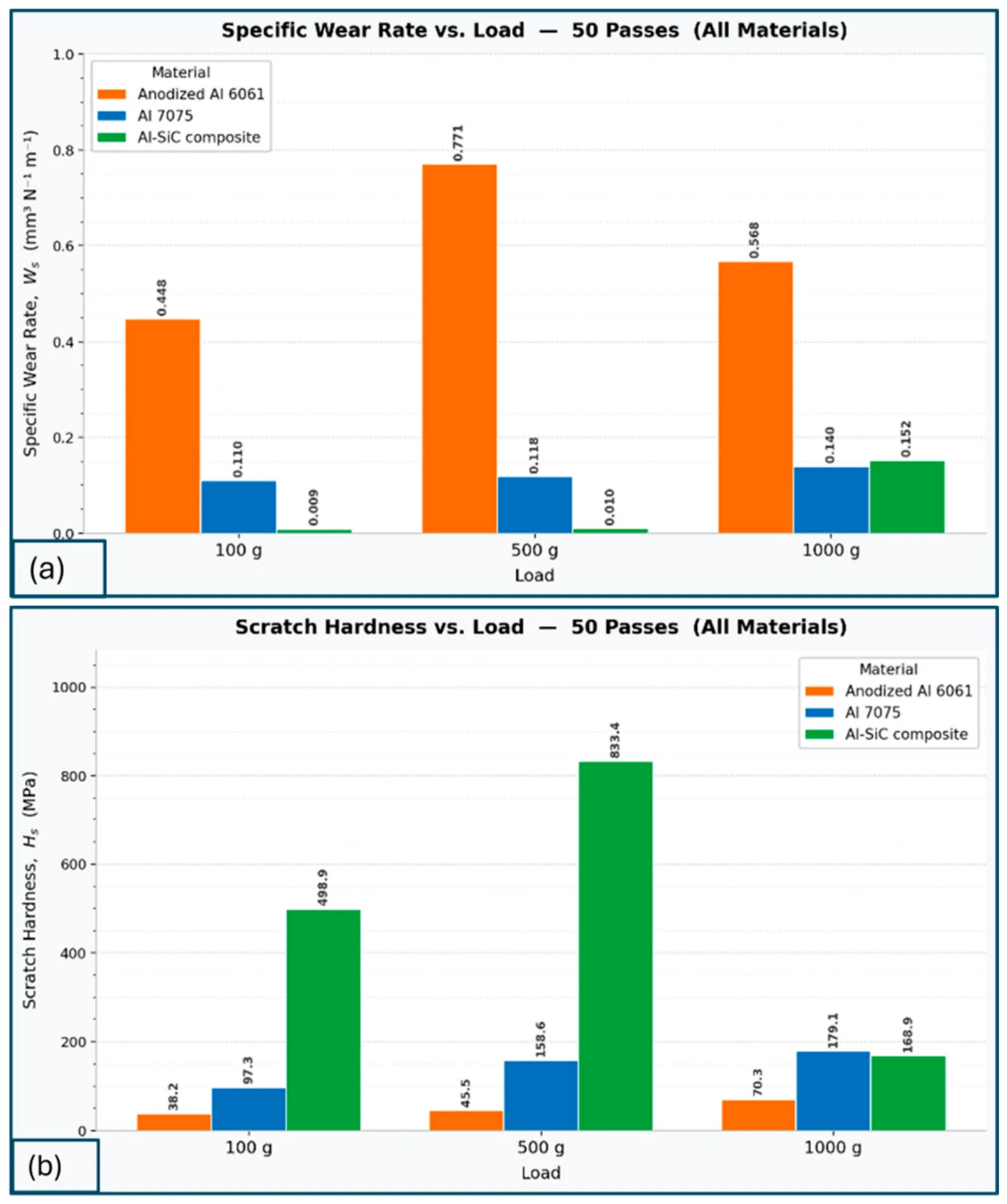
Wear response of materials with varying load; (**a**) Specific wear rate; (**b**) Scratch hardness.

The specific wear rate (Ws) in Figure 12a shows the volume removed normalized by load and sliding distance, following the Archard formulation [29,30]. Scratch hardness (Hs) (Figure 12b), computed from the applied normal load and the scratch groove width and represents the material’s resistance to plastic penetration under a moving indenter. Archard’s law predicts an inverse relationship between wear volume and hardness, so a mirror-image relationship between the two charts is expected, as precisely shown by the data: the material ranking in Figure 12b is almost exactly inverted in Figure 12a.

**Figure 13:**
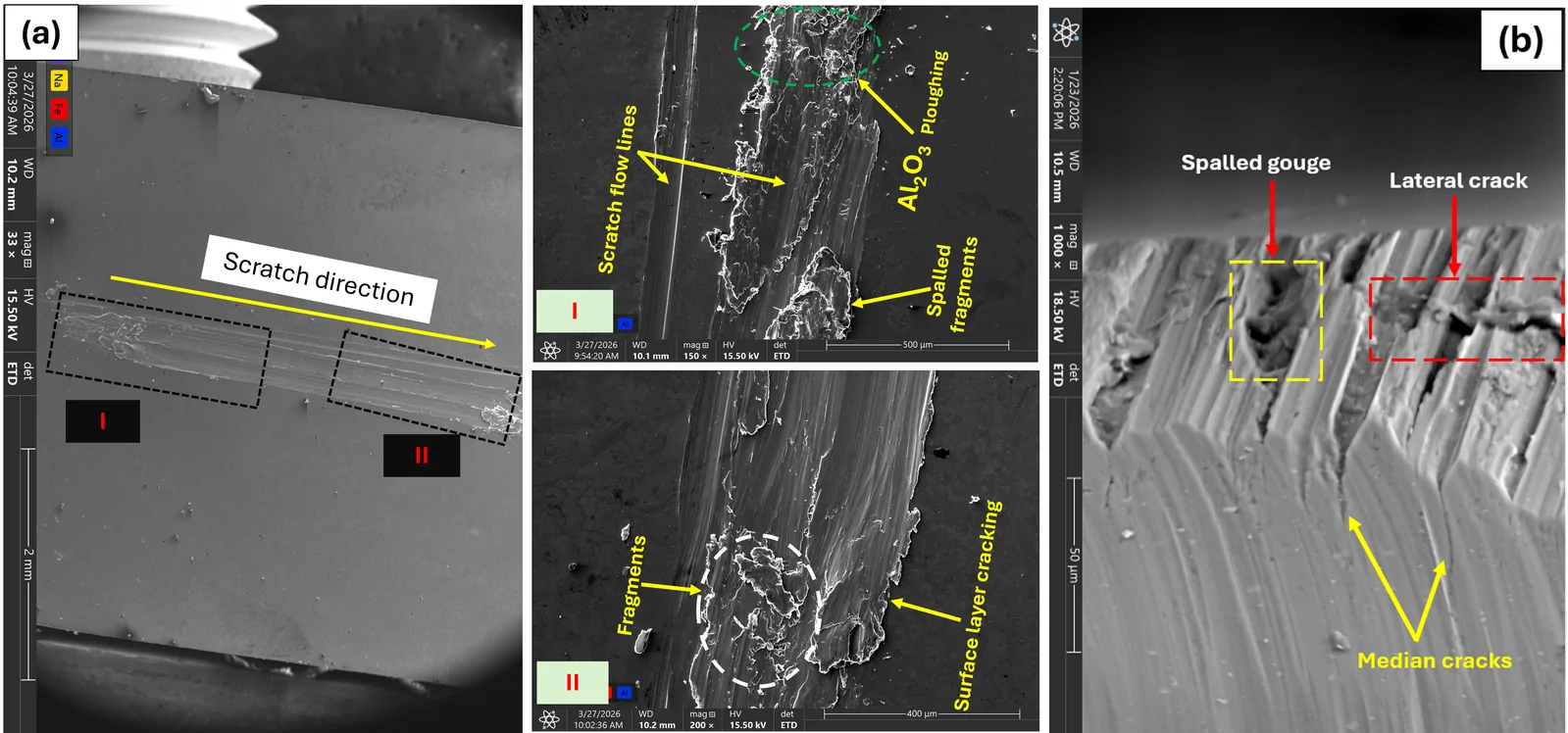
Observed layer cracking and fragments spallation in Anodized AA6061-T6; (**a**) Scratched surface (I and II indicate magnifications in (**a**)); (**b**) Scratch cross-section.

The anodized sample shows the highest W_S_ at every load (0.448 → 0.771 → 0.568 mm^3^ N^−1^ m^−1^) and the lowest HS (38–70 MPa). An intact anodic Al2O3 film has an intrinsic H of roughly 3–5 GPa, far above anything measured. A measured HS of only ~40–70 MPa demonstrates that by 50 passes the film has been fully breached and the indenter is ploughing the soft 6061 substrate, likely mixed with fractured oxide debris. The mechanism is well documented: anodic films are hard but brittle, and on a compliant Al substrate they fail by through-thickness cracking and spallation (Figure 13) once the plastic zone in the substrate undercuts the film. The classic “thin hard coating on soft substrate” (eggshell) failure mode discussed extensively in coating tribology [31]. Once spalled, the angular Al_2_O_3_ fragments act as hard third-body abrasives trapped in the contact, which accelerates wear of the exposed substrate rather than protecting it. This explains why the anodized sample wears faster than untreated AA7075-T6: the coating’s failure products are themselves an abrasive medium. Several studies on multipass scratch and sliding of anodized Al report exactly this transition from protective behavior at low load/few cycles to severe abrasive wear after film perforation.

The observed cracking and spallation of the anodized layer are consistent with the thin hard coating on a compliant substrate model [31]. Plastic deformation of the Al substrate promotes fracture and delamination of the brittle anodized layer.

The non-monotonic peak at 500 g (0.771) followed by a lower value at 1000 g (0.568) is plausibly a debris-compaction effect: at the highest load, fractured oxide can be compacted and smeared into a partially protective mechanically mixed layer (MML), a phenomenon repeatedly observed in aluminum tribo-systems as mentioned in Rigney’s work on mechanically mixed layers [32] and Li & Tandon, Wear, on Al MMCs [33].

**Figure 14:**
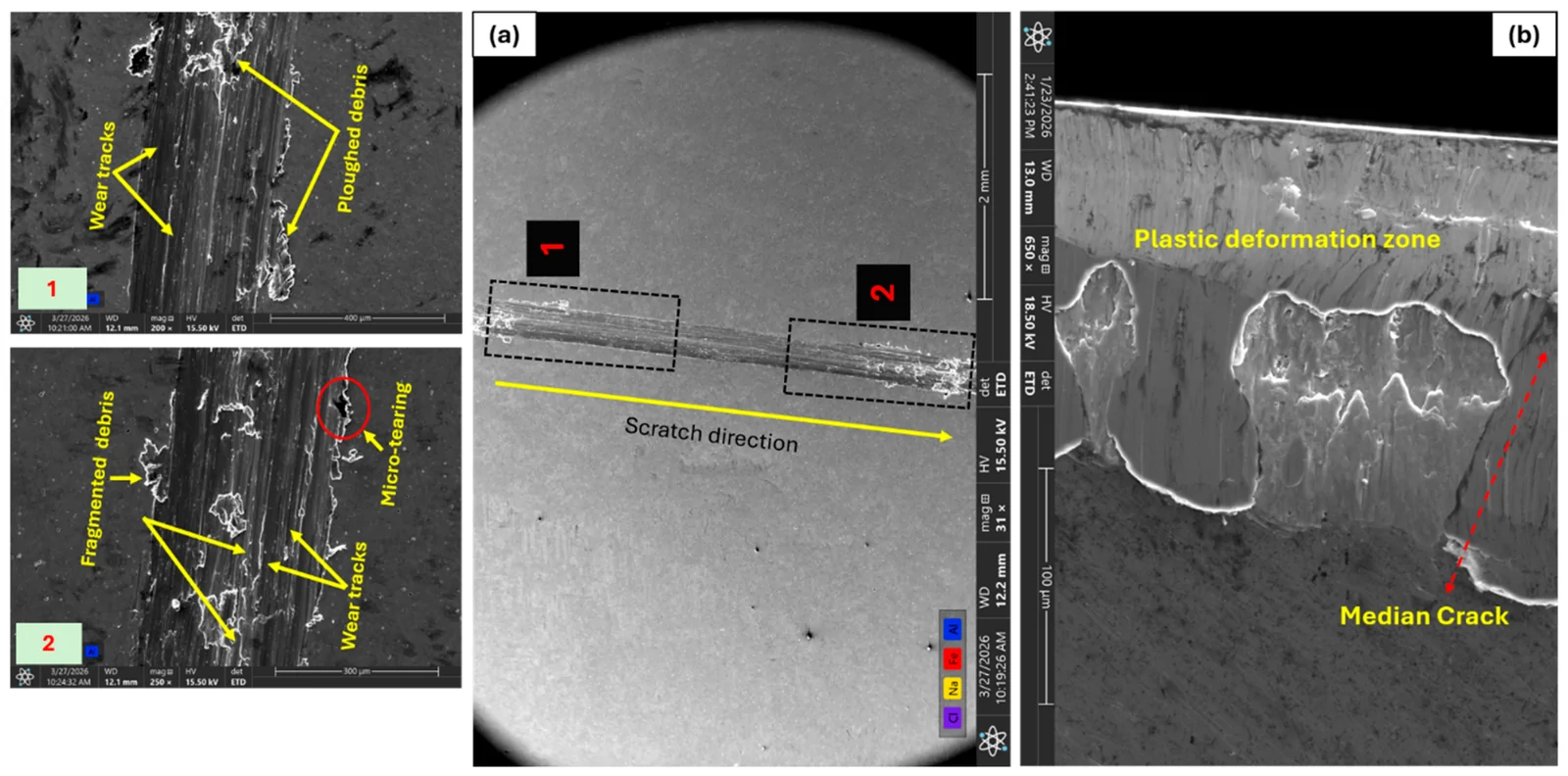
Scratch damage observed in AA7075-T6; (**a**) Scratched surface (Regions 1 and 2 indicate magnifications in (**a**)); (**b**) Scratch cross-sectional features.

AA7075-T6 behaves like a textbook precipitation-hardened Al alloy. W_S_ rises moderately with load (0.110 → 0.118 → 0.140), and H_S_ rises concurrently (97 → 159 → 179 MPa). Two points merit comment; first, the fact that W_S_ (already normalized by load) still increases with load indicates the onset of a mild-to-severe wear transition, consistent with the framework of Archard & Hirst [30]: as contact pressure rises, subsurface plastic strain accumulates and delamination-type damage in accordance with Suh’s delamination theory [34] supplements ploughing (Figure 14a). Second, the increase in H_S_ with load reflects strain hardening of the groove subsurface under repeated passes after 50 traversals, the deformed layer beneath the scratch is heavily work-hardened (Figure 14b), and at higher loads the indenter samples this hardened material more deeply. This multipass hardening effect is a recognized feature of repeat-pass scratch testing on ductile metals.

The Al-15 vol.% SiC T4 composite is the most interesting case. At 100 g and 500 g it is exceptional: WS of 0.009–0.010 (an order of magnitude below AA7075-T6, nearly two below the anodized sample) with HS of 499 and 833 MPa. This is the expected regime in which the hard SiC particulates (~25 GPa) carry the contact load and shield the Al matrix from direct plastic ploughing—the classical protective mechanism established for Al-SiC composites by Alpas and Zhang [35], who mapped mild-wear regimes where reinforcement suppresses wear by 1–2 orders of magnitude relative to the unreinforced alloy.

**Figure 15:**
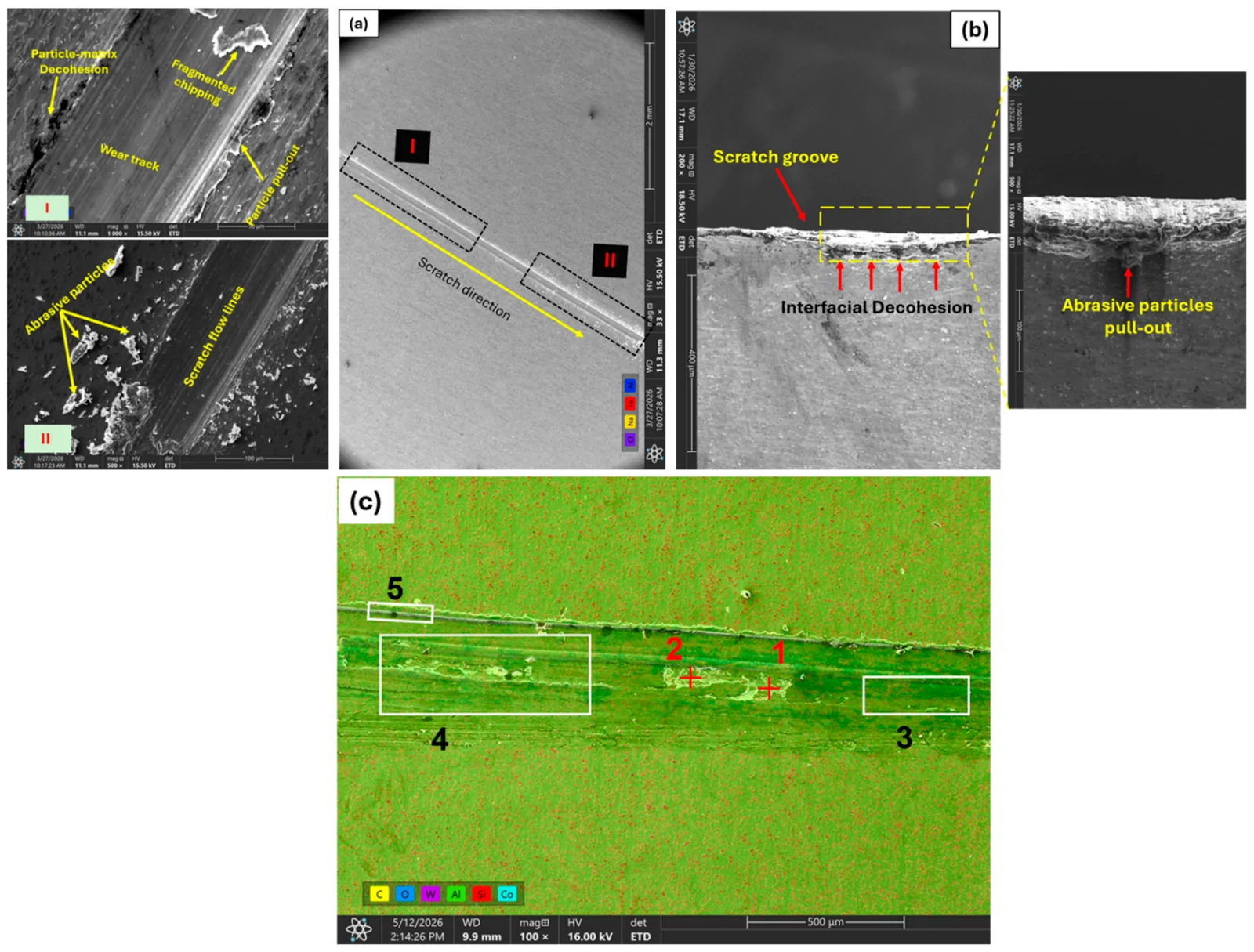
Scratch damage in Al-15 vol.% SiC T4; (**a**) Scratched surface (Regions I and II indicate magnifications in (**a**)); (**b**) Scratch cross-section.; (**c**) EDX analysis of scratch groove (+ and rectangles indicate the analyzed points and regions, respectively, while the numbers correspond to their respective identification numbers).

At 1000 g, however, the picture inverts sharply: W_S_ jumps ~15-fold to 0.152, essentially converging with AA7075-T6 (0.140), and HS collapses from 833 to 169 MPa. This is the signature of exceeding the critical load for particle fracture and decohesion (Figure 15a,b). Alpas and Zhang explicitly identified this transition: above a critical stress, SiC particles fracture and/or debond, the fragments are incorporated as abrasive third bodies (Figure 15c), and the composite loses its wear advantage, behaving like (or worse than) the matrix alloy. Similar critical-load transitions are reported across the Al-MMC literature (e.g., Sannino & Rack [36] in their review of dry sliding of particulate-reinforced Al). HS drop at 1000 g is the mechanical counterpart of the same event once particles fracture and pull out under the moving stylus, the measured penetration resistance reverts toward the matrix value.

The EDX analysis shown in Figure 15c (Table 3) confirms the presence of Si-rich particles (Points 1 and 3), identifying them as SiC reinforcement fragments within the Al matrix, while Regions 4 and 5 are predominantly Al-rich, representing the exposed matrix after wear. The elevated C content at Point 3 (48.7 wt.%) together with the detectable Si confirms fractured or detached SiC particles embedded within the wear track. These observations support the proposed wear mechanism that, above a critical applied stress, SiC particles fracture and/or debond from the matrix. The resulting fragments become abrasive third bodies, promoting three-body abrasion, accelerating material removal, and diminishing the wear resistance of the composite, causing it to behave similarly to or even worse than the unreinforced Al matrix.

### 4.5. Hertzian Indentation Analysis

The Hertzian-type indentation test was performed as a single comparative loading cycle for each material under identical conditions. Accordingly, the resulting load–displacement curves and observations presented in the related figures represent typical responses obtained under the specified testing and analytical conditions rather than averaged results. These results are intended to illustrate the comparative surface and subsurface mechanical response and associated features, rather than provide a statistically representative assessment of experimental repeatability or scatter, as independent duplicate datasets were not acquired.

**Figure 16:**
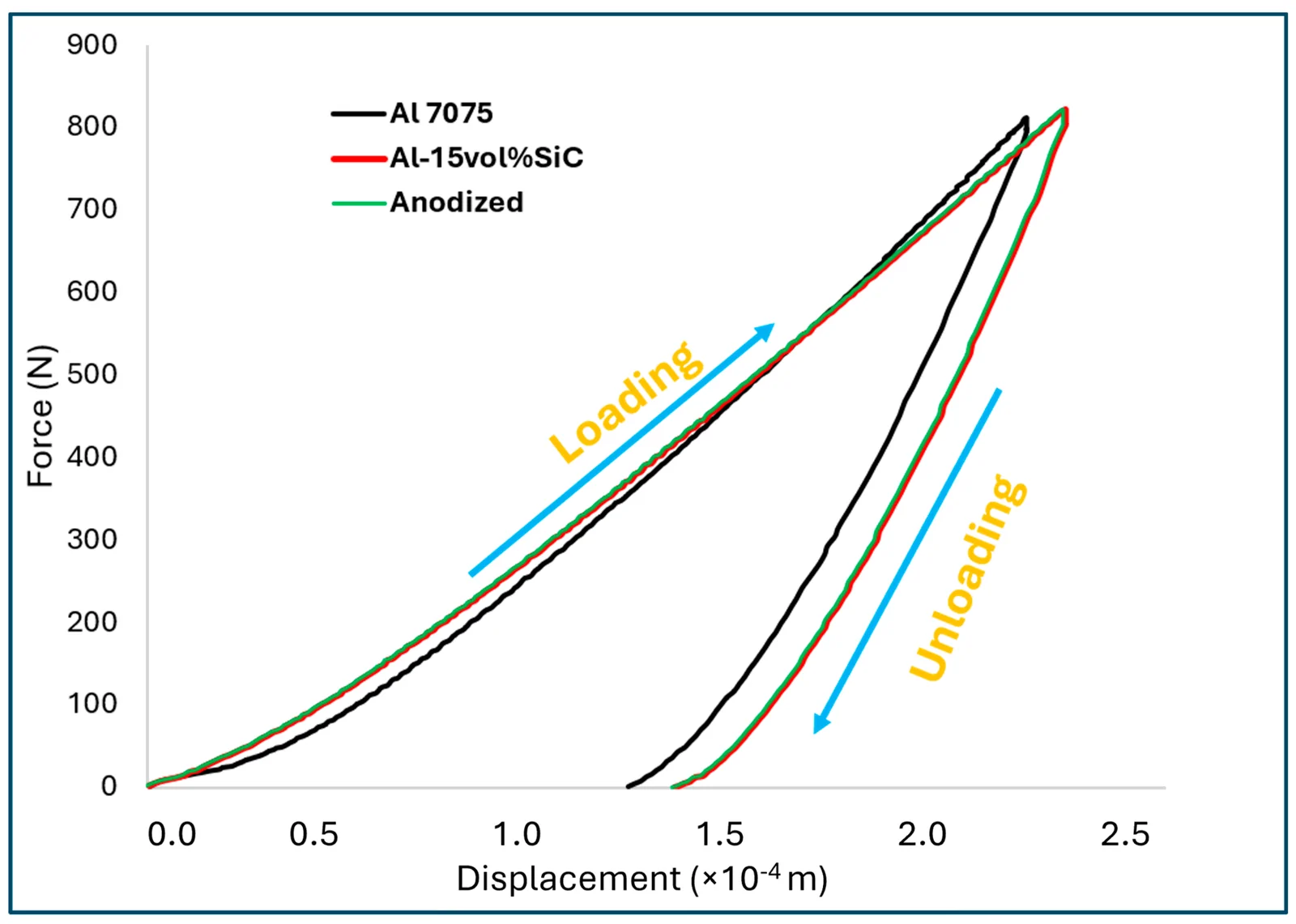
Load/Unloading-Displacement behavior of materials.

The load–displacement curves (Figure 16) from the Hertzian-type indentation critically project the contact stiffness and deformation resistance of AA7075-T6, Anodized AA6061-T6, and the Al-15 vol.% SiC T4 composite. The overall trend shows clear differences in how each material accommodates load during indentation, which can be directly related to their microstructural characteristics. At the early stage, the loading curves reveal that the Al-15 vol.% SiC T4 composite and anodized AA6061 exhibit a steeper initial loading response than AA7075-T6, indicating greater resistance to indenter penetration. This behavior reflects their higher near-surface stiffness and Hs, requiring a greater applied load to achieve the same indentation depth. In the case of the Al-15 vol.% SiC T4 composite, the hard SiC reinforcement particles effectively impede localized plastic deformation and increases the effective modulus and H of the material by constraining matrix deformation and enhancing load transfer, while the hard anodic Al_2_O_3_ layer on AA6061 provides a mechanically robust surface that resists indentation. Conversely, the relatively gentler loading response of AA7075-T6 indicates lower resistance to indentation under the same loading conditions, resulting in greater penetration for a given applied load.

**Figure 17:**
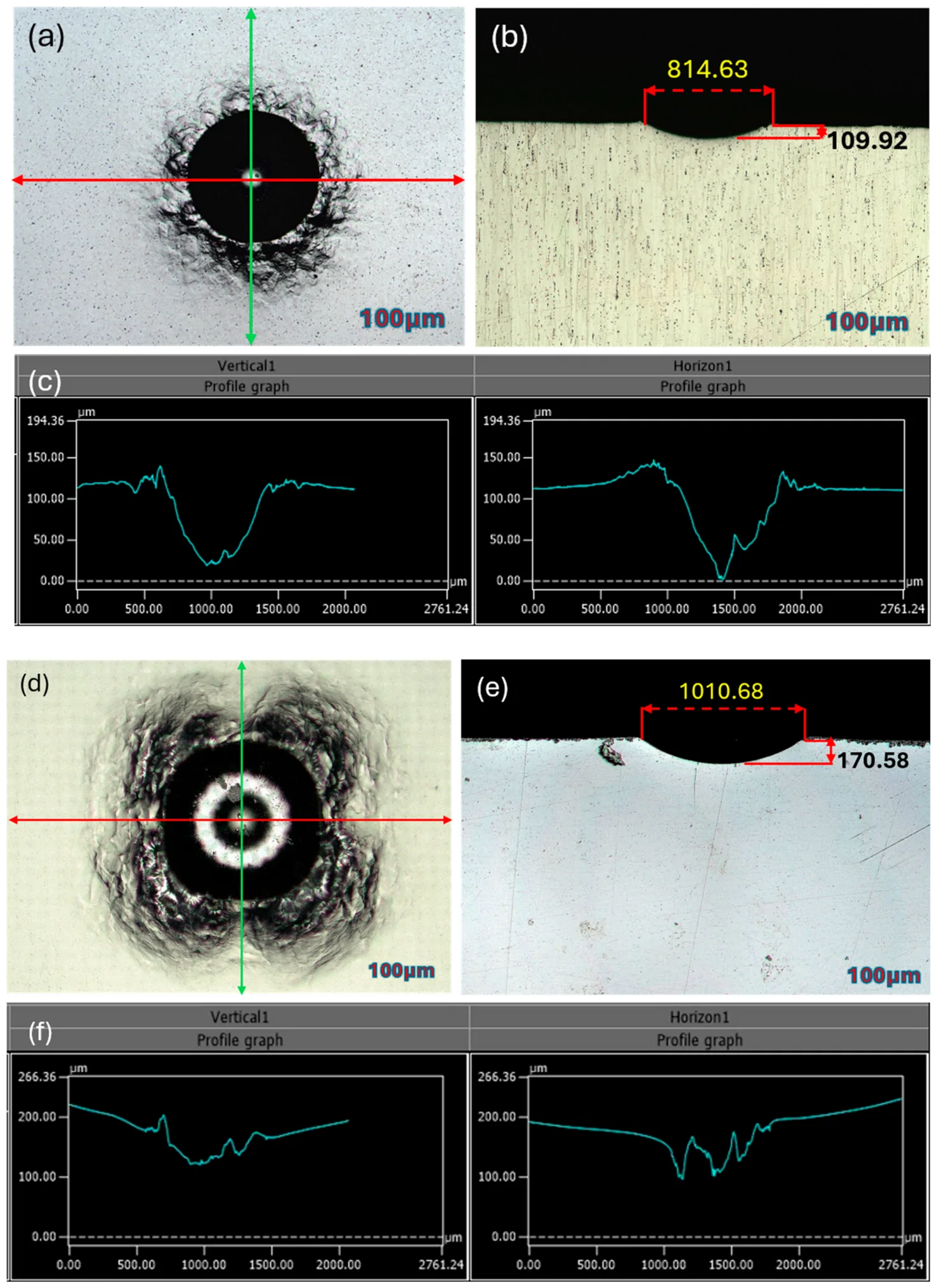

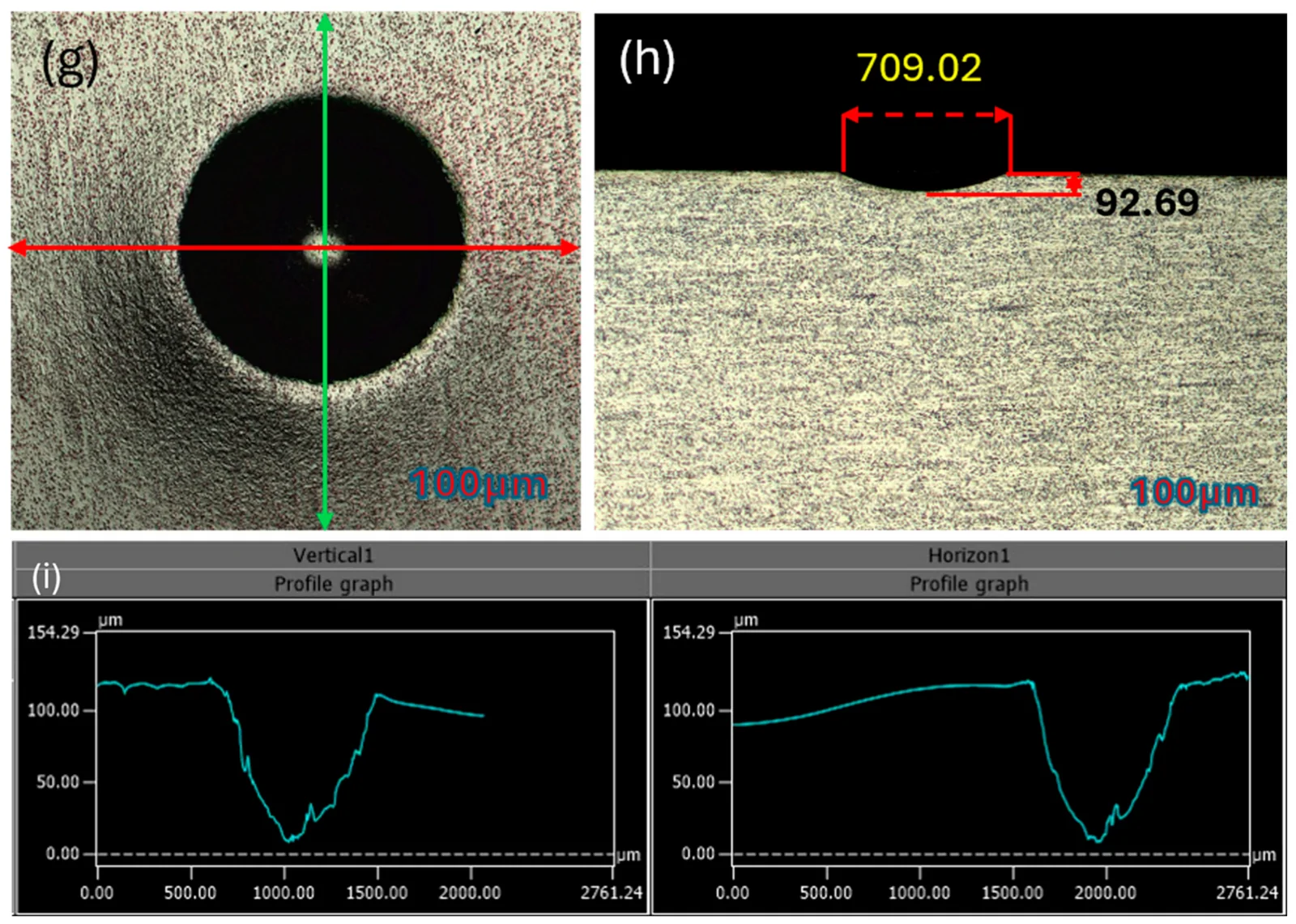
Optical micrographs of indented surface, cross-section, and surface profiles of specimens (The red and green lines indicate the positions of horizontal and vertical surface profiles across the indents, respectively); (**a**–**c**) AA7075-T6; (**d**–**f**) Anodized AA6061-T6; (**g**–**i**) Al-15 vol.% SiC T4.

The analysis of the three indented specimens focused on their surface morphology, surface profiles, and cross-sectional characteristics (Figure 17), particularly the depth and width of the indents. CLSM examination did not reveal any visible subsurface cracking at the cross-sectional level within the resolution of the technique. Detailed observations and measurements for each specimen are presented in the subsequent paragraphs.

CLSM did not resolve subsurface cracks at the inspected cross-sections within the spatial resolution of the technique. This does not indicate that cracks were absent. SEM examination at higher magnification revealed localized edge cracking, delamination, and microcracks. The methods are therefore complementary: CLSM was used for indentation geometry, while SEM was used to identify fine-scale damage.

The indented surface of AA7075-T6 (Figure 17a) shows a well-defined circular indentation surrounded by a pronounced deformation zone. The central dark region represents the permanent plastic impression formed at the point of indenter contact where the highest compressive stresses were applied. Around this cavity, an irregular halo of radially distributed features is observed, indicating material pile-up and localized plastic flow resulting from lateral displacement of material during indentation. These outward-extending patterns also suggest the presence of localized slip and shear deformation, where plastic strain was accommodated along preferred crystallographic slip systems typical of precipitation-hardened Al alloys. The rough morphology at the indentation boundary reflects intense plastic deformation and possible minor microfracturing of surface asperities; however, the absence of well-defined radial cracking indicates that the deformation response is predominantly ductile.

The cross-section of the AA7075-T6 indentation (Figure 17b) exhibited a width of 814.63 µm and a depth of 109.92 µm, indicating moderate penetration under the applied load. The smooth, bowl-shaped cavity reflects deformation dominated by plastic yielding within the Al matrix, where dislocation motion allows the material to accommodate the contact stresses. This subsurface response corresponds with the surface morphology, where material displacement around the indentation produced the observed pile-up and deformation halo. The absence of significant subsurface cracking further indicates that the indentation response is governed primarily by ductile plastic flow.

The surface profile of AA7075-T6 (Figure 17c) shows a relatively smooth and symmetric indentation cavity in both perpendicular directions across the indentation, as shown. The profiles indicate a well-defined central depression with gradual slopes on either side, suggesting uniform plastic deformation beneath the indenter. The surrounding region exhibits limited irregularity, indicating that the material deformed in a comparatively homogeneous manner. This behavior is consistent with the high strength but still ductile nature of AA7075-T6, where the material accommodates the applied load primarily through plastic flow rather than brittle fracture or severe surface disruption. The smooth profile curves and moderate depth also suggest that the material maintained structural continuity around the indentation without significant surface cracking or fragmentation.

The indented surface of the Anodized AA6061-T6 (Figure 17d) exhibited features similar to those observed in AA7075-T6, with a central indentation surrounded by a deformation zone; however, the surrounding region displayed more intense and irregular ripple-like features. Mechanistically, these ripples arise from the mechanical mismatch between the hard, brittle anodized oxide layer and the ductile Al substrate. Under indentation, the oxide layer experiences high localized stresses that promote microcracking, fragmentation, and partial delamination, while the underlying Al accommodates the load through plastic deformation. This difference in deformation behavior leads to stress redistribution and localized strain accumulation, causing the oxide layer to fracture and displace in a ripple-like pattern around the indentation boundary. Consequently, the observed morphology reflects a coupled deformation mechanism involving brittle fracture of the anodized coating and plastic flow of the substrate, resulting in the more pronounced irregularities compared to the homogeneous plastic deformation observed in AA7075-T6.

The cross-section of Anodized AA6061-T6 (Figure 17e) specimen showed the largest indentation with a width of 1010.68 µm and a depth of 170.58 µm, indicating greater penetration and lateral deformation. This behavior is associated with the mechanical mismatch between the brittle anodized oxide layer and the ductile Al substrate. Under loading, the oxide layer undergoes localized cracking and fragmentation, after which the softer substrate accommodates the load through plastic deformation. This combined response explains both the increased indentation dimensions and the ripple-like irregularities observed on the surface, which arise from oxide fracture and substrate displacement.

The profile obtained from Anodized AA6061-T6 (Figure 17f) shows an evidently different response characterized by pronounced irregularities within the indentation cavity. The profiles, of the two perpendicular directions across the indentation impression, display multiple fluctuations and localized peaks and valleys within the depression region. These irregular features correspond to the fractured or fragmented regions visible in the optical images. The anodized layer, which is a hard but brittle Al_2_O_3_ coating, likely fractured under the concentrated contact stresses during indentation. This results in localized collapse and uneven deformation of the surface. Consequently, the indentation profile appears rough and discontinuous compared with the smoother curves observed in AA7075-T6. The deeper and more complex indentation geometry suggests that the brittle oxide layer experienced cracking and spallation, followed by localized deformation of the softer Al substrate beneath it.

The indented surface of the Al-15 vol.% SiC T4 composite (Figure 17g) exhibited greater resistance to indentation, as evidenced by the absence of intense plastic deformation around the contact region. Instead, the indentation boundary showed slight outward or upward protrusion, which can be attributed to limited localized material displacement during loading. Mechanistically, the presence of hard SiC reinforcement particles within the Al matrix increases the composite hardness and load-bearing capacity, thereby restricting extensive plastic flow typically observed in monolithic Al alloys. Under indentation, the applied load is partially transferred and distributed through the stiff SiC particles, which act as barriers to dislocation motion and inhibit large-scale plastic deformation. This constraint promotes localized elastic-plastic response and minor material uplift at the indentation edges, rather than pronounced extensive surface damage. Consequently, the surrounding region appears relatively clean and undisturbed, indicating that the reinforcement effectively stabilizes the matrix and limits deformation to a confined zone beneath the indenter.

The Al-15 vol.% SiC T4 composite exhibited the smallest indentation dimensions (Figure 17h), with a width of 709.02 µm and a depth of 92.69 µm, demonstrating improved resistance to indentation. The presence of hard SiC reinforcement particles enhance the stiffness and hardness of the composite, limiting dislocation motion and restricting plastic deformation beneath the indenter. Consequently, deformation remains highly localized, producing a shallower and narrower cavity.

The surface profiles of the Al-15 vol.% SiC T4 (Figure 17i) corroborated those observed in (Figure 17h). The two perpendicular profiles across the indenter impression exhibited a broader and smoother surface depression followed by a gradual recovery toward the original surface level. The surrounding surface remains comparatively stable with minimal large-scale irregularities. This behavior is likely influenced by the presence of hard SiC reinforcement particles. These particles restrict extensive plastic flow of the matrix and may cause localized stress concentrations beneath the indenter. As a result, the matrix undergoes constrained deformation while the reinforcement particles resist penetration, leading to a well-defined but relatively steep indentation geometry.

**Figure 18:**
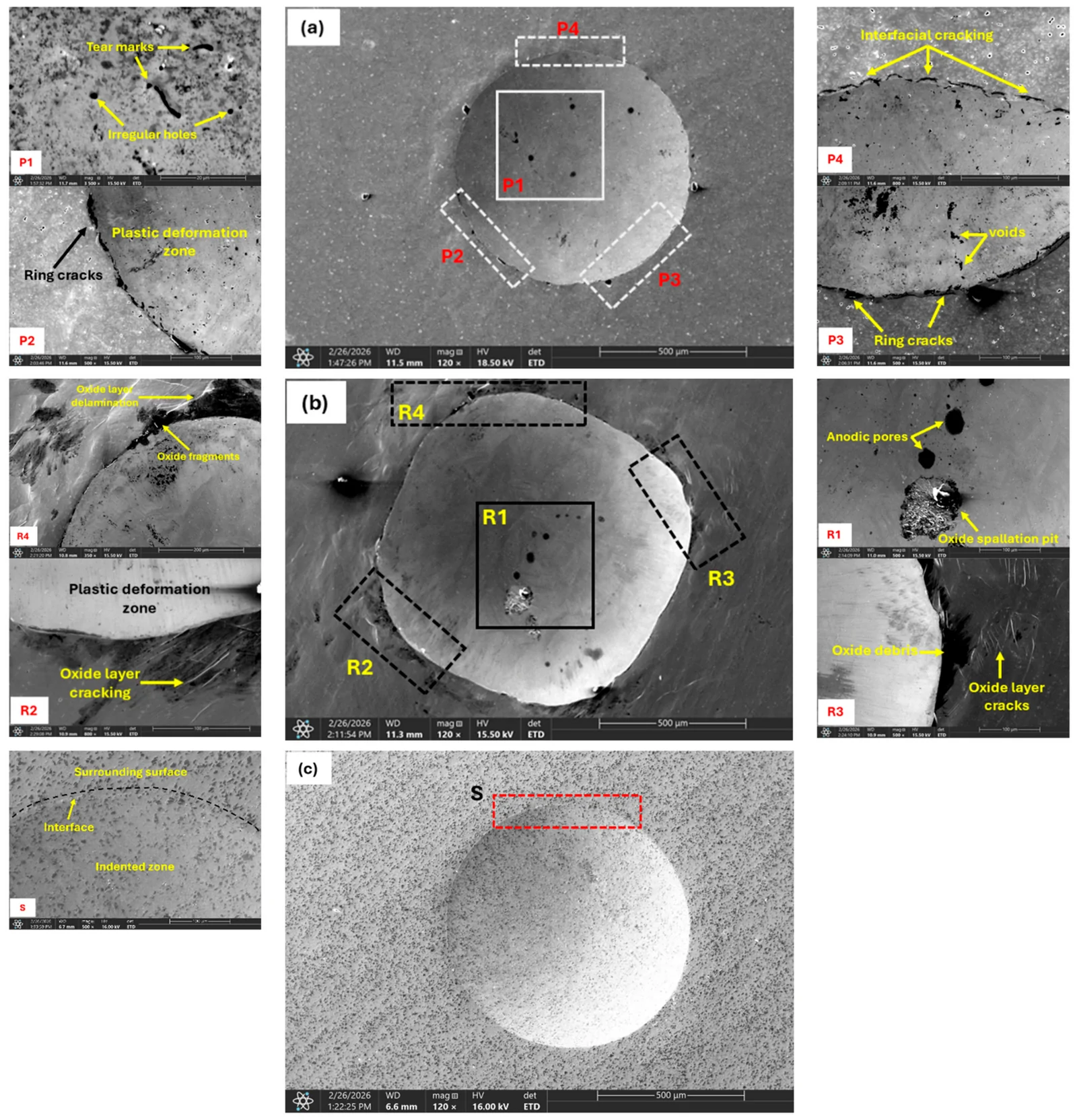
Indented surface analysis under SEM; (**a**) AA7075-T6; (**b**) Anodized AA6061-T6; (**c**) Al-15 vol.% SiC T4.

SEM was further employed to examine the surfaces and cross-sections of the indented specimens at higher magnifications (Figure 18), enabling the identification of features that were not resolved under CLSM. The surface of the AA7075-T6 specimen (Figure 18a) revealed pronounced tear marks and irregular void-like holes within the indented region (P1). These features are indicative of severe localized plastic deformation produced during indentation, where the high contact stresses beneath the indenter promote intense shear flow of the material. Such plastic flow can lead to localized tearing and the formation of micro-voids as the material is displaced and stretched beyond its local ductility limits. At the indent-matrix interface (P2 and P3), ring cracking accompanied by small voids was observed around the plastically deformed zone. Hertzian-type ring cracking typically forms due to tensile stresses that develop at the perimeter of the contact zone as the indenter penetrates and the surrounding material elastically recovers during unloading [37,38]. This stress concentration at the boundary of the plastically deformed region can initiate circumferential cracks and micro-void formation. Additionally, the uppermost portion of the indent exhibited interfacial cracking or tearing with a zigzag and irregular morphology (P4). This behavior likely results from strain localization and shear instability near the surface, where the material experiences complex combinations of compressive and tensile stresses during loading and unloading, leading to discontinuous fracture along weakened microstructural paths [39,40].

The surface of the Anodized AA6061-T6 specimen (Figure 18b) displayed characteristic anodic pores and localized oxide spallation pits within the indented region (R1), reflecting the inherent porosity and brittle nature of the anodic oxide layer. At the indent-matrix boundary (R2 and R3), cracking of the oxide layer was observed, accompanied by detached debris from these fractures. This occurs because the hard, brittle oxide layer cannot accommodate the plastic deformation of the underlying Al substrate, resulting in tensile and shear stresses that exceed the fracture strength of the oxide, causing it to crack and produce debris. Additionally, evidence of delamination of the oxide layer from the substrate was observed (R4), leaving behind oxide fragments. This delamination arises from the mechanical mismatch between the stiff oxide coating and the more ductile aluminum underneath. During indentation, the substrate undergoes plastic flow, generating interfacial stresses at the oxide-metal interface that overcomes the adhesive strength of the oxide, causing it to lift, crack, and separate from the underlying material. These features collectively highlight the interplay between brittle coating behavior and ductile substrate deformation under concentrated contact loading.

Consistent with the earlier CLSM observations at lower magnification, the Al-15 vol.% SiC T4 composite (Figure 18c) maintained its rigidity without any visible cracking. This behavior can be attributed to the presence of SiC reinforcement particles, which enhance hardness and impede plastic flow, effectively distributing the applied load and minimizing stress concentrations. As a result, both surface and cross-sectional analyses confirm the composite’s resistance to cracking under the applied indentation. Region ‘S’ on the indented surface of the Al-15 vol.% SiC T4 composite revealed a clear boundary between the indent zone and the surrounding matrix, highlighting the localized nature of the deformation.

**Figure 19:**
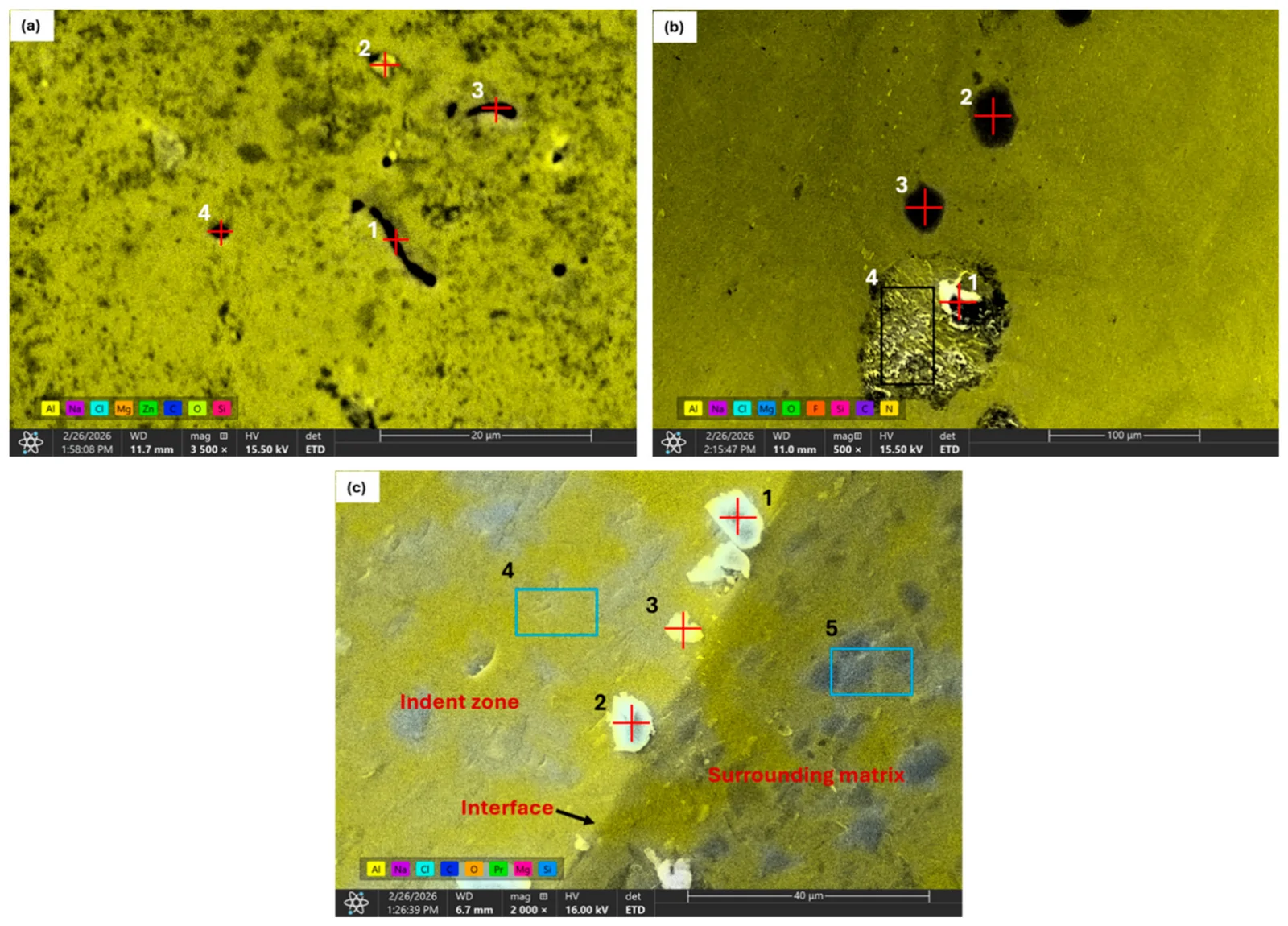
Energy Dispersive X-ray (EDX) analysis on specimens’ surfaces; (**a**) AA7075-T6 (Figure P1); (**b**) Anodized AA6061-T6 (Figure R1); (**c**) Al-15 vol.% SiC T4 interface.

EDX analysis of the AA7075-T6, Anodized AA6061-T6, and Al-15 vol.% SiC T4 specimens revealed distinct compositional characteristics that are consistent with their respective microstructures and Hertzian indentation responses (Figure 19, Table 4). The elemental compositions in Table 4 were obtained directly from an automated EDX point/region scanning and quantification and are reported as measured weight percentages. As independent replicate analyses were not performed, no statistical error ranges are assigned to these values. For AA7075-T6, Al remained the dominant element (43.3–65.2 wt.%), with localized enrichments of Zn, Si, Fe, and O. The Zn-rich regions are consistent with the alloy’s strengthening phases, whereas Fe- and Si-rich areas indicate the presence of intermetallic particles and inclusions. Oxygen detected at selected locations suggests localized surface oxidation. These microstructural heterogeneities contribute to local variations in hardness and stiffness, influencing stress distribution and plastic deformation beneath the indenter.

The Anodized AA6061-T6 specimen was similarly dominated by Al (0.2–67.0 wt.%), with oxygen detected primarily at Point 1 (11.2 wt.%) and Region 4 (3.7 wt.%), confirming the presence of the anodized oxide layer. Small amounts of Mg (0.2–0.8 wt.%) are consistent with the nominal alloy composition. Variations in oxygen concentration suggest differences in oxide layer thickness or partial penetration of the coating during indentation. As a result, the indentation response reflects the combined behavior of the hard, brittle anodized layer and the underlying ductile Al substrate, producing localized variations in deformation.

In contrast, the Al-15 vol.% SiC T4 composite exhibited the greatest compositional heterogeneity. Aluminum content varied from 3.2 to 69.8 wt.%, while Si reached 48.2 wt.% in reinforcement-rich regions. The concurrent detection of C (13.7–28.5 wt.%) confirms the presence of SiC particles, whereas minor oxygen indicates localized surface oxidation. The heterogeneous distribution of hard SiC particles within the softer Al matrix governs the indentation response by restricting local plastic deformation and promoting stress concentrations at the particle–matrix interface. Consequently, the composite exhibited localized irregularities in the indentation profile compared with the more homogeneous responses of the monolithic Al alloys. Overall, the EDX results demonstrate that the observed Hertzian indentation behavior is strongly influenced by the local chemical composition and microstructural heterogeneity of each material.

**Figure 20:**
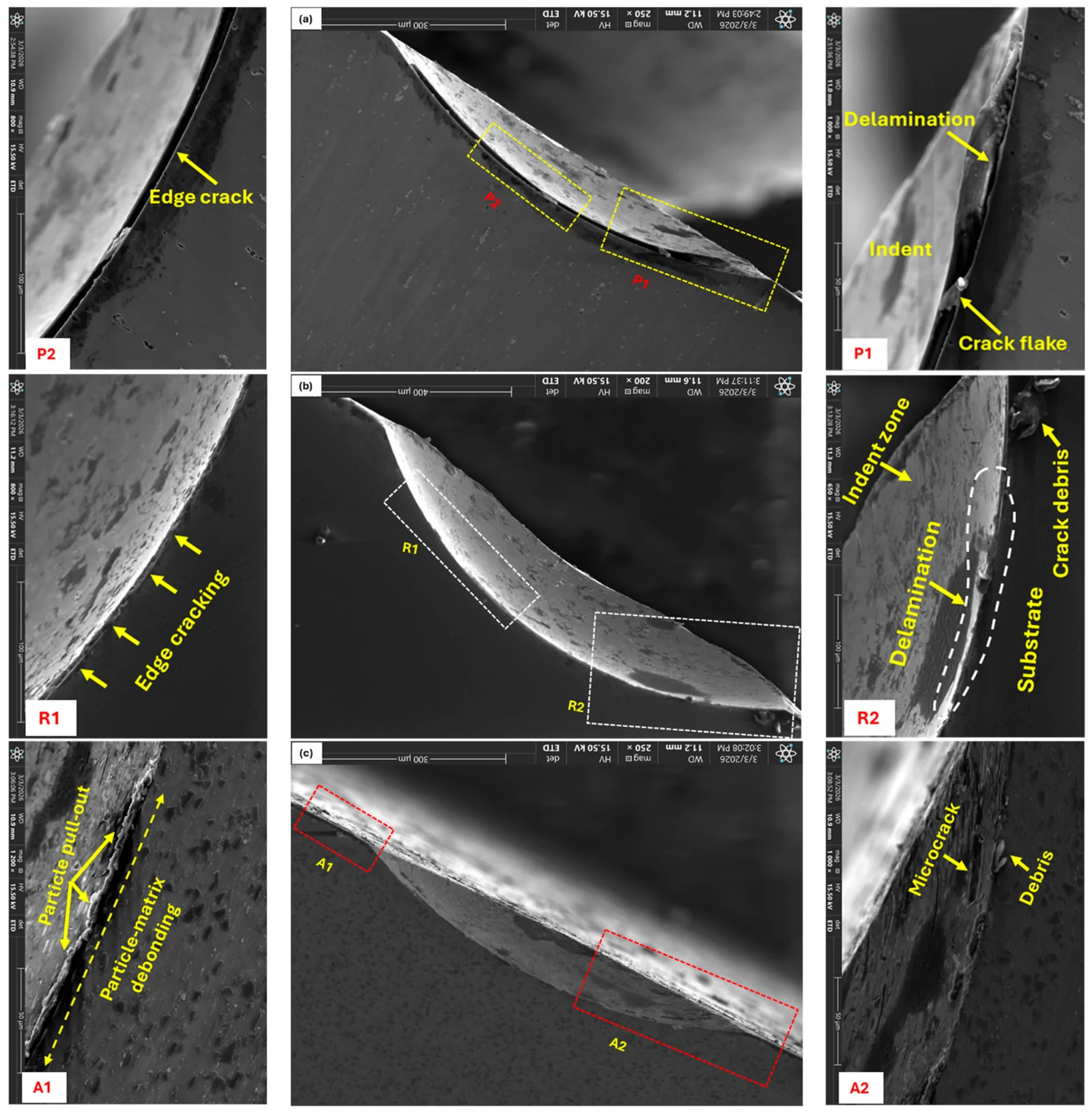
Cross-sectional evaluation of Indented specimens under SEM; (**a**) AA7075-T6; (**b**) Anodized AA6061-T6; (**c**) Al-15 vol.% SiC T4 composite.

The cross-sectional SEM observations (Figure 20) reveal distinct deformation and damage mechanisms for the three indented specimens, reflecting the influence of microstructure, interfacial integrity, and local stress distribution beneath the indenter.

For the AA7075-T6 specimen (Figure 20a), the presence of edge cracking (P2), crack flakes, and delamination (P1) along the indent-subsurface boundary indicates localized brittle fracture initiated by the high stress concentration generated during indentation. AA7075-T6 is a high-strength precipitation-hardened alloy whose strengthening precipitates increase hardness but reduce the material’s ability to plastically accommodate localized deformation [28,41]. Consequently, the compressive stresses beneath the indenter, coupled with tensile stresses at the indentation edges during unloading, promote crack initiation along the indent boundary. The observed crack flakes and subsurface delamination suggest that once cracks nucleated, they propagated along relatively weak microstructural paths such as precipitate-matrix interfaces or shear-localized zones, resulting in the partial separation of material layers.

In the Anodized AA6061-T6 specimen (Figure 20b), the occurrence of edge cracking (R1) accompanied by cracking debris and delamination reflects the mechanical mismatch between the hard anodized oxide layer and the more ductile Al substrate. As mentioned, the anodized surface is primarily brittle with significantly higher hardness but limited fracture toughness. Under indentation loading, the oxide layer experiences constrained deformation and accumulates tensile stresses, particularly near the indentation edges. This promotes crack initiation within the oxide layer, and the resulting fragments appear as debris surrounding the crack sites. Delamination (R2) observed beneath the indent further indicates partial separation of the anodic layer from the substrate, likely driven by interfacial shear stresses and the difference in elastic-plastic response between the brittle oxide coating and the ductile Al base [42,43].

In contrast, the Al-15 vol.% SiC T4 composite (Figure 20c) exhibits material pull-out, interfacial debonding (A1), and minor microcracks (A2), suggesting that the dominant damage mechanism is governed by the reinforcement-matrix interaction. The presence of hard SiC particles restricts plastic flow of the Al matrix, leading to localized stress concentrations at the particle-matrix interfaces during indentation. When the applied stress exceeds the interfacial bonding strength, debonding occurs, resulting in particle pull-out and the formation of interfacial voids [44]. The relatively limited microcracking observed indicates that the composite matrix retains some ability to plastically accommodate deformation, thereby reducing extensive crack propagation compared with the monolithic alloy or brittle anodized layer.

**Figure 21:**
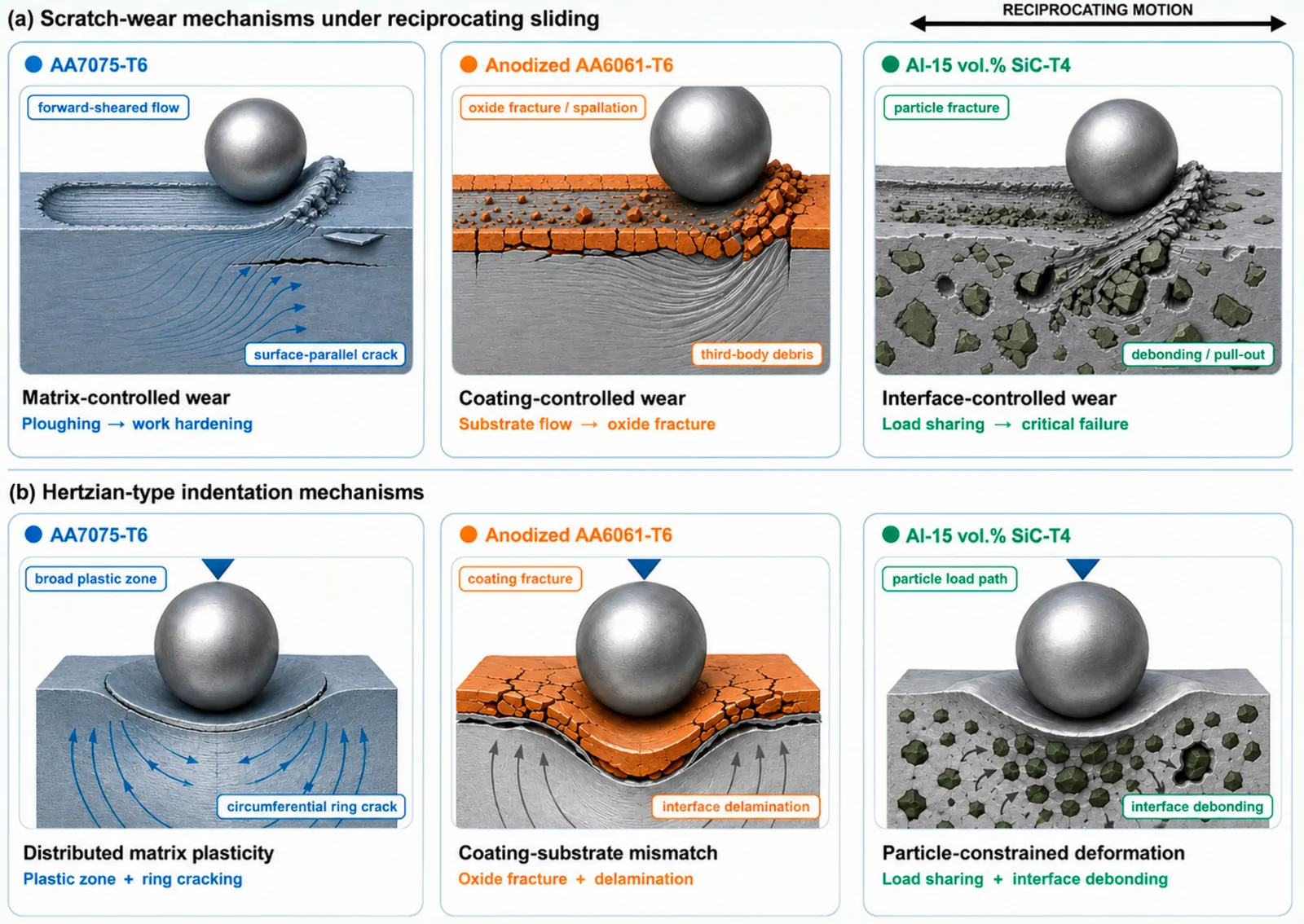
Schematic summary of dominant (**a**) scratch-wear and (**b**) Hertzian-type indentation damage mechanisms in AA7075-T6, Anodized AA6061-T6, and Al-15 vol.% SiC-T4. Schematic not to scale.

Based on the surface and cross-sectional observations discussed above, the dominant wear and Hertzian-type indentation mechanisms for the three investigated materials are summarized schematically in Figure 21.

In relation to the present Hertzian-type indentation results, the variation in deformation and damage behavior observed among AA7075-T6, Anodized AA6061-T6, and Al-15 vol.% SiC T4 can be attributed to differences in how each material accommodates stress contours [45]. For AA7075-T6, the relatively ductile matrix facilitates a more distributed stress field, promoting plastic deformation and resulting in smooth indentation profiles with minimal cracking. In divergence, the Anodized AA6061-T6 system exhibits a mechanically heterogeneous structure, where the stiff and brittle oxide layer modifies the stress distribution by amplifying tensile stresses near the surface, resulting in the shattered surface seen under CLSM as shown in Figure 17d. This leads to the initiation of radial cracking and potential coating delamination under increasing loads. For the Al-15 vol.% SiC T4 composite, the presence of rigid SiC reinforcements constrain matrix deformation and alters the stress contours, producing localized stress concentrations at particle-matrix interfaces. These localized stresses contribute to interfacial debonding and microcrack formation despite the material’s overall high resistance to penetration.

## 5. Conclusions

In this study, three distinct classes of Al-based systems; AA7075-T6, Anodized AA6061-T6-T6 (oxide layer with an average thickness of 8.71 µm), and Al-15 vol.% SiC-T4 (particle size range of 3.1–8.3 µm) commonly employed in structural and tribological applications, were systematically investigated under wear and indentation loading conditions. The results demonstrate that although all three materials are Al-based systems, their resistance to localized deformation and sliding damage is strongly governed by differences in microstructure, surface condition, and reinforcement mechanisms. Major findings from this study are highlighted below:

1. Nanoindentation highlighted the strong influence of microstructure, surface/coating architecture, and reinforcement phase on the depth-dependent mechanical response of materials. AA7075-T6 exhibited a stable bulk H response associated with its precipitation-strengthened matrix, whereas Anodized AA6061-T6-T6 showed a pronounced coating-substrate transition, reflecting the strong influence of the hard anodic Al_2_O_3_ layer at shallow depths and the underlying Al substrate at greater penetration. The Al-15 vol.% SiC T4 composite demonstrated comparable H stabilization but a relatively higher E, attributable to the stiffness and load-transfer contribution of the SiC reinforcement.
2. The wear responses were strongly load-dependent. Al-15 vol.% SiC exhibited superior wear resistance at lower loads due to effective load bearing by SiC particles, but its performance deteriorated sharply at 1000 g because of particle fracture/decohesion and third-body abrasion. AA7075-T6 showed moderate wear resistance with progressive work hardening, whereas Anodized AA6061-T6-T6 exhibited the highest wear due to brittle coating failure, substrate exposure, and abrasive oxide debris.
3. Hertzian-type indentation outcome was strongly governed by the microstructure and mechanical heterogeneity of the three Al-based systems. AA7075-T6 exhibited predominantly plastic deformation with localized cracking interfacial debonding, while Anodized AA6061-T6-T6 showed the greatest penetration and surface damage due to brittle oxide fracture, spallation, and coating-substrate mismatch. In contrast, Al-15 vol.% SiC T4 exhibited the smallest indentation and greatest resistance to penetration, although localized particle-matrix debonding and pull-out occurred under the concentrated stresses. Thus, the indentation damage mechanisms transitioned from matrix-dominated plastic deformation in AA7075-T6, to coating-substrate fracture and delamination in Anodized AA6061-T6, and particle-matrix interfacial damage in the SiC-reinforced composite.

## Figures and Tables

**Table 1:** Elemental compositions of investigated materials.

	Element (Weight %)								
Material	Al	Cr	Cu	Fe	Mg	Mn	Zn	Ni	Si
AA7075-T6	90.00	0.21	1.29	0.24	2.56	0.03	5.47	0.01	0.53
Anodized AA6061-T6	97.95	0.06	0.20	0.23	0.83	0.07	0.01	0.01	0.76
Al-15 vol.% SiC T4	77.16	0.003	3.45	0.13	1.14	0.04	0.002	0.02	1.35

**Table 2:** Measured hardness of specimens.

	Rockwell Hardness (HRB)			
Material	AA6061-T6		AA7075-T6	Al-15 vol.% SiC T4 Composite
	Substrate	Anodized surface		
Average hardness	50.2 ± 0.2	55.8 ± 1.9	85.4 ± 0.7	81.8 ± 2.0

**Table 3:** EDX outcome of analyzed scratched surface of Al-SiC shown in Figure 15c.

	Weight %				
Element	Point 1	Point 2	Point 3	Region 4	Region 5
C	15.5	13.3	48.7	9.2	10.3
O	5.4	7.0	8.9	3.7	6.5
Al	58.5	77.9	35.8	76.3	69.9
Si	19.6	0.8	6.1	10.0	12.2
Co	0.0	0.0	0.0	0.0	0.0
W	1.0	1.0	0.5	0.8	1.1

**Table 4:** Summary of EDX point/region analyses (weight %) for the surfaces of AA7075-T6, Anodized AA6061-T6, and Al-15 vol.% SiC T4 samples (P and R denote point and region respectively).

Material	Analysis Location	C	O	Mg	Al	Si	Zn	Fe
AA7075-T6	P1	–	12.2	0.9	45.9	14.5	2.1	0.0
	P 2	–	0.0	0.0	54.7	0.0	2.4	14.5
	P 3	–	11.3	0.9	43.3	20.0	0.0	0.0
	P 4	–	0.3	1.3	65.2	0.0	4.3	0.0
Anodized AA6061-T6	P 1	–	11.2	0.8	30.1	–	–	–
	P 2	–	0.3	0.2	0.2	–	–	–
	P3	–	0.0	0.2	43.3	–	–	–
	R 4	–	3.7	0.2	67.0	–	–	–
Al-15 vol.% SiC T4	P 1	28.5	17.7	–	3.2	2.2	–	–
	P2	14.9	1.5	–	5.6	1.0	–	–
	P3	23.2	1.7	–	31.4	1.7	–	–
	R 4	13.7	3.2	–	69.8	12.0	–	–
	R 5	15.6	1.4	–	33.4	48.2	–	–

Note: Only the major elements related to the discussed test outcomes are reported; therefore, the listed values do not necessarily sum to 100 wt.%.

## Data Availability

The original contributions presented in the study are comprised in the article, further inquiries can be directed to the corresponding author.

## Abbreviations

The following abbreviations are used in this manuscript:

Al_2_O_3_	Aluminum Oxide
Al-SiC	Aluminum-Silicon Carbide Composite
CLSM	Confocal Laser Scanning Microscopy
CSM	Continuous Stiffness Measurement
E_r_	Reduced Modulus
E	Elastic Modulus (Young’s Modulus)
EDX	Energy Dispersive X-ray Spectroscopy
H	Hardness
HCl	Hydrochloric Acid
HF	Hydrofluoric Acid
HNO_3_	Nitric Acid
H_s_	Scratch Hardness
ICDD	International Centre for Diffraction Data
ICP-OES	Inductively Coupled Plasma Optical Emission Spectroscopy
MEMS	Micro-Electro-Mechanical Systems
MML	Mechanically Mixed Layer
Mg_2_Si	Magnesium Silicide
MMCs	Metal Matrix Composites
PDF	Powder Diffraction File
Ra	Arithmetic Average Surface Roughness
SEM	Scanning Electron Microscopy
XRD	X-ray Diffraction
YS	Yield Strength
